# Frequency-Encoded Magnetic Resonance Imaging with Dynamic Radio Frequency Field Gradients

**DOI:** 10.1038/s42005-026-02686-5

**Published:** 2026-05-18

**Authors:** Sai Abitha Srinivas, Antonio D. Glenn, Mark A. Griswold, William A. Grissom

**Affiliations:** 1 Department of Biomedical Engineering, Case Western Reserve University, Cleveland, Ohio, USA; 2 Department of Radiology, Case Western Reserve University, Cleveland, Ohio, USA

## Abstract

Magnetic Resonance Imaging is the gold standard for soft tissue diagnostic imaging but relies on expensive, failure-prone, and loud magnetic field gradients for spatial encoding. Radiofrequency field gradients are a promising alternative, yet they have never achieved frequency encoding, the technique that allows an image to be acquired continuously in milliseconds and without which scan times would increase by orders of magnitude. Here we show that the Bloch-Siegert shift can apply a spatial frequency gradient simultaneously with signal recording, without displacing magnetization from the plane where it generates signal. Combined with an injection transformer based simultaneous transmit-and-receive system that prevents the strong encoding field from overwhelming the weak tissue signal, this approach produces images on a 47.5 mT low-field scanner equivalent in quality to those from conventional gradient encoding. This work enables smaller, quieter, and less expensive scanners without compromising scan time or diagnostic image quality, with particular promise for expanding access to imaging in resource-limited settings.

## Two-Sentence Summary

MRI scanners depend on large, noisy magnetic gradient coils that limit their accessibility worldwide. Here we show that radiofrequency field gradients can perform frequency encoding with simultaneous transmit and receive, producing images matching conventional quality and enabling smaller, lower-cost MRI systems.

## Introduction

Magnetic Resonance Imaging (MRI) generates high-resolution diagnostic images of the body's internal anatomy with excellent soft tissue contrast. Lauterbur^1^ first demonstrated the fundamental idea underpinning MRI when he superimposed a gradient magnetic field onto a uniform background ($B_{0}$) magnetic field, to establish a mapping between spatial position and the temporal resonance frequency of tissue protons. In this experiment, the gradient magnetic field was applied simultaneously during signal recording, a technique that is now referred to as frequency encoding. Frequency encoding is an essential element that makes MRI fundamentally feasible; without it, MRI scans can take at least two orders of magnitude more time, much too long to be used clinically. Today, frequency encoding is universally implemented using $B_{0}$ gradient coils and associated support and driving systems. However, these systems are a source of many of MRI’s weaknesses including their bulk, their complex design^2^, their cost^3^, and their active cooling requirement which limits space in the bore and makes MRI challenging in claustrophobic populations. $B_{0}$ gradient encoding requires a significant amount of power, to the point where MRI scanners are dominant energy consumers in a hospital. $B_{0}$ gradients are the sole source of the loud acoustic noise^4,5^ in MRI which necessitates ear protection. Indeed, United States Food and Drug Administration (FDA) approval of MRI is contingent on the use of hearing protection from $B_{0}$ gradient noise^6^. $B_{0}$ gradients are also prone to breakage due to the powerful Lorentz forces they generate. Finally, their switching speed is limited by eddy currents. In recent years, there has been a resurgence in the development of low-field MRI scanners, to increase access to MRI^3,7,8^, achieve point of care applications^9–15^, and enable new image-guided therapies^16,17^. While moving to low-field strengths reduces magnet cost, power, and complexity, the fundamental limitations of $B_{0}$ gradient systems persist since gradient performance requirements still demand high power consumption, substantial cost, and complex engineering. $B_{0}$ gradient-based systems also pose different and additional challenges at these field strengths, including $B_{0}$ field drift due to the heat the gradients generate in permanent magnet-based systems^18^ and large concomitant gradient terms that scale as $1/B_{0}$^19^ and cause image distortions^20^.

Radiofrequency (RF) gradient systems have been proposed as an alternative to $B_{0}$-based gradient systems^21–27^. RF gradients offer several advantages, including compactness, quiet operation, faster switching with minimal cooling, lower cost, and compatibility with commodity RF amplifiers. Furthermore, they provide additional degrees of freedom for spatial encoding, especially for non-traditional low-$B_{0}$-strength magnet geometries^25^. At low $B_{0}$ strengths RF gradients also alleviate concomitant gradient errors, and they are not limited by RF-induced tissue heating, since Specific Absorption Ratio (SAR), a measure of RF-induced tissue heating in MRI, depends on the square of $B_{0}$ field strength^5,28,29^. However, frequency encoding has never been achieved using RF gradient systems. One roadblock to achieving RF gradient frequency encoding has been the lack of a mechanism to simultaneously apply spatially varying frequency shifts to magnetization while keeping it mostly in the transverse plane so that it continuously generates a detectable electromotive force while accruing spatially dependent phase. This is a challenge because the RF magnetic field that interacts with magnetization is also in the transverse plane, so previous RF gradient encoding sequences^21,23,24,30,31^ have had to fully rotate magnetization out of the transverse plane, preventing simultaneous spatial encoding and signal recording. Since any RF-based frequency encoding would require simultaneous transmission and reception, the second roadblock has been the lack of simultaneous transmit and receive systems with sufficiently high isolation to prevent the transmitted RF encoding pulse from overwhelming the several-orders-of-magnitude weaker proton signal and causing irreversible hardware damage. Both challenges must be overcome for RF gradient encoding to become a feasible alternative to $B_{0}$ gradient systems.

This work reports the first implementation, to the best of our knowledge of frequency-encoded MRI using RF gradients, achieved by leveraging the Bloch-Siegert shift^32^ to spatially manipulate proton frequencies. When used in conjunction with an RF coil whose magnetic field varies spatially, the Bloch-Siegert shift provides a mechanism to apply a spatially varying frequency shift to magnetization without significantly tipping it out of the transverse plane, solving the mechanistic problem. The challenge of simultaneously applying an RF encoding pulse while recording signal was overcome with a novel simultaneous transmit and receive system based on an injection transformer, which allowed high-power RF transmission for spatial encoding while preserving the sensitivity to detect faint proton signals. Deployed on a 47.5 mT MRI scanner^33,34^ with an optimized RF gradient coil, this system successfully produced images with visible contrast, demonstrating compatibility with conventional MRI pulse sequences. This work paves the way for a new generation of MRI scanners with multiple advantages in size, cost, robustness, and patient comfort.

## Results

### Frequency Encoding with RF Gradients

**Figure 1:**
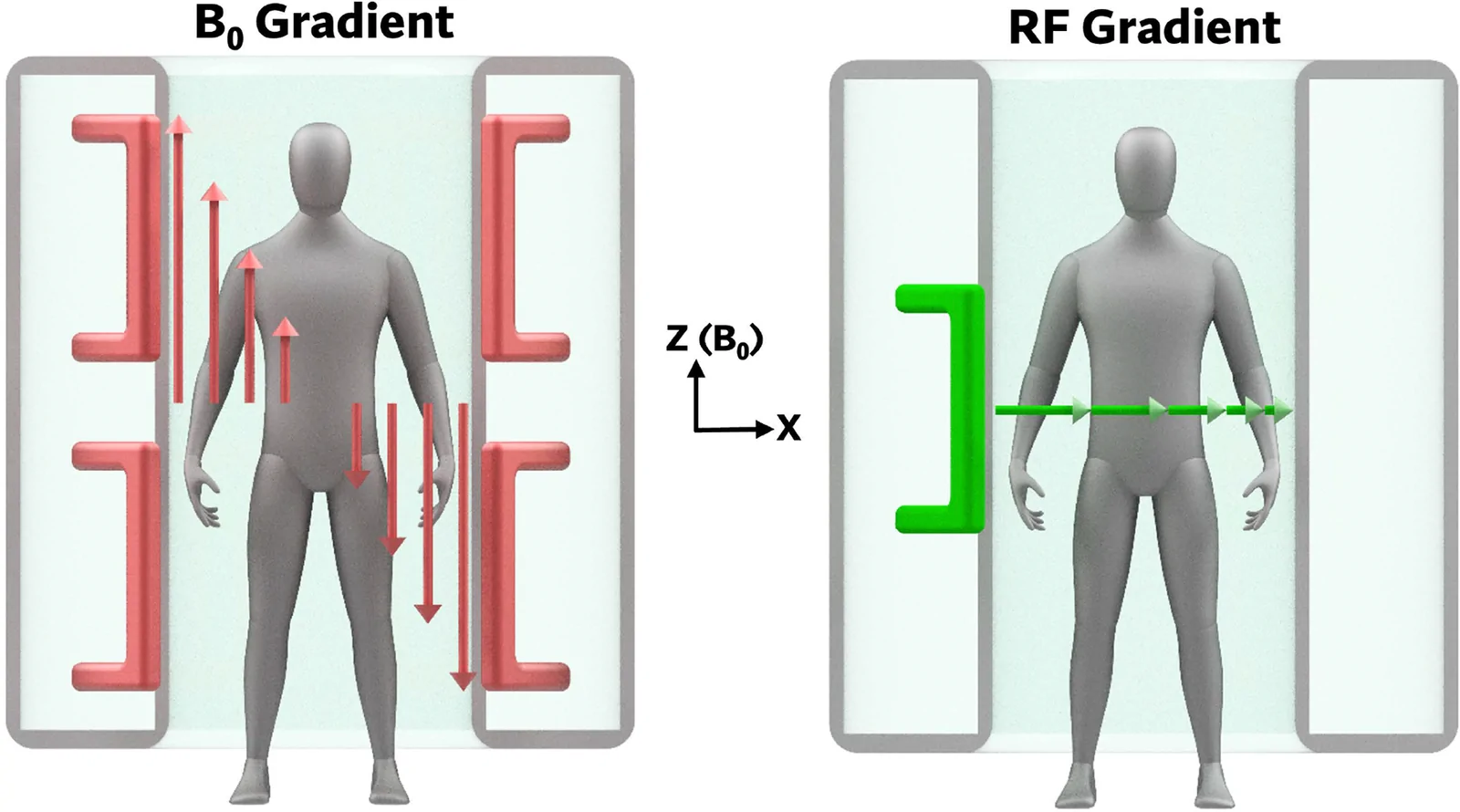
Illustration of B_0_ (main magnetic field) and RF gradient coils in an MRI scanner for spatial encoding a patient’s left-right (x) direction, wherein the scanner’s B_0_ field is along the z-direction. A B_0_ gradient coil (left) produces a spatial variation in the z-component of the static magnetic field B_0_. In contrast, an RF gradient coil produces spatially varying x/y RF magnetic field components. Arrow length represents magnetic field amplitude.

Conventional MRI uses $B_{0}$ gradient coils to induce linear variations in field strength. As illustrated in Figure 1, these coils generate magnetic fields that add to or subtract from the z-directed $B_{0}$ magnetic field, generating a gradient in that field along their respective spatial encoding axes. In contrast, RF gradient coils produce spatially varying transverse (x/y) magnetic field components, in the same plane as the excited proton magnetization being imaged.

We used the Bloch-Siegert (BS) shift^32^ to produce spatially varying shifts in proton magnetization precession frequencies using an RF gradient coil. First described in 1940, the BS shift is a systematic frequency shift that arises from an off-resonant RF pulse, which has historically been regarded as a nuisance that affects the accuracy of frequency sensitive nuclear magnetic resonance measurements. The BS shift produced by an RF pulse is proportional to the square of the magnetic field amplitude ($B_{1}$) generated by the RF coil carrying the pulse, and inversely proportional to the pulse’s offset from the Larmor frequency. Hence, when an RF pulse with a frequency offset is produced by an RF gradient coil, a spatially varying frequency gradient is applied to the object subjected to the coil’s field. If the RF gradient coil’s field is a square-root function of space, the frequency gradient will be linear. The BS shift can thereby be harnessed for spatial encoding, and we and others have used the BS shift to localize signal via spatially selective excitation^35–37^ and RF gradient phase encoding^26,27,38^.

**Figure 2:**
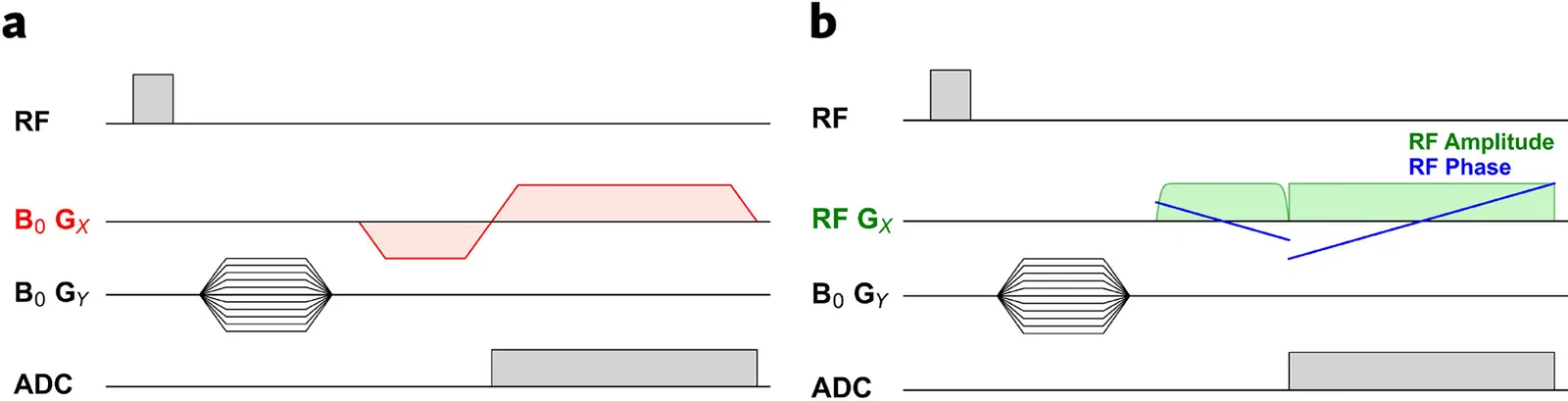
Illustration of gradient-recalled echo (GRE) pulse sequences using B_0_ (main magnetic field) and RF gradient frequency encoding. a) A conventional 2D GRE pulse sequence using B_0_ gradients, wherein G_x_ is the frequency encoding gradient with pre-phasor and readout pulses, and G_y_ is the phase encoding gradient. b) The same pulse sequence but with RF gradient frequency encoding via the Bloch-Siegert shift, where G_x_ is now an RF gradient with off-resonant pre-phasor and readout pulses.

Figure 2a shows a conventional 2D Gradient Recalled Echo (GRE) sequence with $B_{0}$ gradient frequency encoding⁵. The sequence comprises an excitation pulse, phase encoding along y with a different G_y_ pulse amplitude each repetition time (TR), and frequency encoding along x with fixed pre-phasing and readout pulses. Figure 2b shows the same sequence but with a Bloch-Siegert RF frequency encoding gradient in x. Bloch-Siegert frequency encoding starts with a pre-phasing RF pulse with a negative frequency offset (as reflected by the negative phase slope) that moves the magnetization phase to the most negative coordinate to be sampled in the k_x_-dimension, followed by a readout RF pulse with a positive frequency offset (reflected by the positive phase slope) that moves the magnetization phase to the most positive coordinate while recording signal. This example illustrates how RF frequency encoding can be implemented in a pulse sequence by one-for-one replacement of $B_{0}$ gradient pulses with off-resonant RF pulses. Next, we derive the signal equations for this sequence.

### Signal Equation for RF Frequency Encoding with the Bloch-Siegert Shift

To derive an equation that describes the signals generated by RF frequency encoding using the Bloch-Siegert shift, we first describe the dynamics of transverse magnetization during RF frequency encoding in a time-dependent rotating reference frame whose angular frequency equals the off-resonant encoding RF pulse's frequency. This is the reference frame in which adiabatic RF pulses are designed and analyzed, and it simplifies the geometric analysis of RF frequency encoding. Then we shift reference frames to the conventional reference frame with angular frequency equal to the Larmor frequency, where MR signal equations are conventionally defined.

A frame rotating about the z-axis at the Bloch-Siegert pulse frequency $\Delta \omega_{RF}$ has axes $\left({\overset{ˆ}{x}}^{\prime },{\overset{ˆ}{y}}^{\prime },{\overset{ˆ}{z}}^{\prime }\right)$ that relate to the Larmor frequency frame axes $\left(\overset{ˆ}{x},\overset{ˆ}{y},\overset{ˆ}{z}\right)$ as:

(1) $$\overset{ˆ}{x}={\overset{ˆ}{x}}^{\prime }cos\left(\Delta \omega_{RF}t\right)-{\overset{ˆ}{y}}^{\prime }sin\left(\Delta \omega_{RF}t\right)$$

(2) $$\overset{ˆ}{y}={\overset{ˆ}{x}}^{\prime }sin\left(\Delta \omega_{RF}t\right)+{\overset{ˆ}{y}}^{\prime }cos\left(\Delta \omega_{RF}t\right)$$

(3) $$\overset{ˆ}{z}={\overset{ˆ}{z}}^{\prime },$$

where t=0 is the beginning of the off-resonant RF pulse. In the Bloch-Siegert pulse frequency frame, magnetization that is on-resonant in the Larmor frame experiences an effective magnetic field

(4) $${\vec{B}}_{eff}=B_{1}{\overset{ˆ}{x}}^{\prime }+\frac{\Delta \omega_{RF}}{\gamma }\overset{ˆ}{z},$$

where the spatial dependence of $B_{1}$ is suppressed for brevity. The magnetization precesses about ${\vec{B}}_{\mathrm{eff}}$ at a frequency of

(5) $$\omega_{eff}=\sqrt{{\left(\gamma B_{1}\right)}^{2}+{\left(\Delta \omega_{RF}\right)}^{2}}.$$

**Figure 3:**
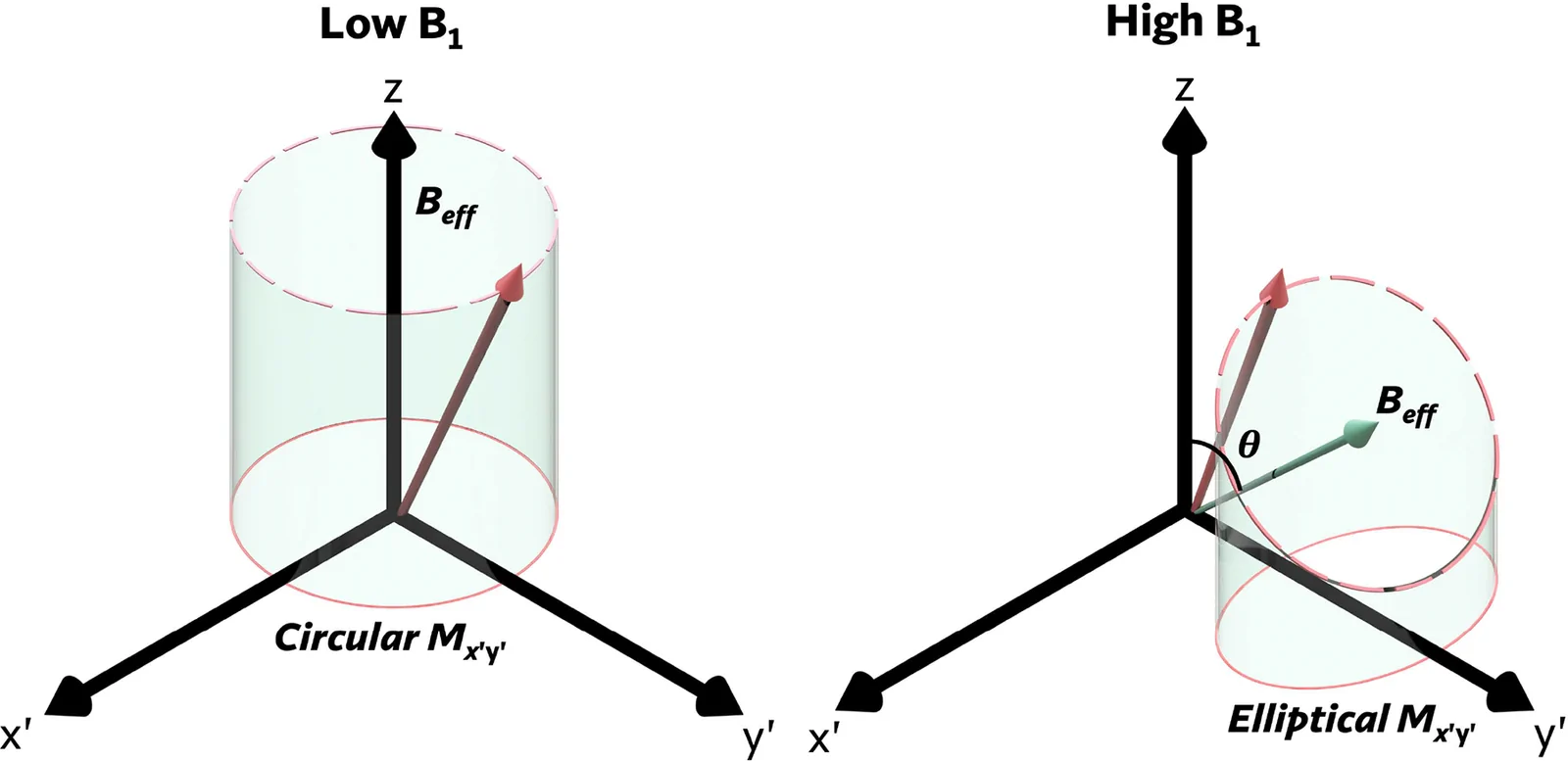
Geometric illustration of RF frequency encoding using the Bloch-Siegert shift in a frame of reference that rotates at the encoding pulse frequency, so that the x’-y’ (transverse) component of the pulse’s effective magnetic field B_eff_ (green arrow) is static, and the pulse has a positive z-field component whose length is proportional to its frequency offset. At a spatial location with low B_1_ (radiofrequency field) amplitude where B_eff_ is nearly exactly along z (tilt angle θ≈0 with respect to the z-axis), the magnetization’s (M_xy_’s) frequency is minimally affected by the pulse, so it traces a circular trajectory and generates a circularly polarized signal. At a location with high B_1_, B_eff_ is tilted away from the z-axis by a larger angle θ, and the magnetization’s trajectory is oblique to the transverse plane, resulting in an elliptically polarized signal.

Consider a magnetization vector $\vec{M}=M_{x}\overset{ˆ}{x}+M_{y}\overset{ˆ}{y}+M_{z}\hat{z}$ defined at the beginning of the off-resonant pulse. Assuming an adiabatic transition, $\vec{M}$ will precess in a cone around ${\vec{B}}_{\mathrm{eff}}$, where the cone is tilted away from the z-axis towards the ${\overset{ˆ}{x}}^{\prime }$ axis by an angle

(6) $$\theta ={tan}^{-1}\frac{\gamma B_{1}}{\Delta \omega_{RF}},$$

which will vary spatially with $B_{1}$. Figure 3 illustrates the cones of rotation for spatial locations with small and large $B_{1}$ amplitudes, corresponding to small and large θ, and small and large $\omega_{\mathrm{eff}}$. Supplementary Movie 1 further illustrates the magnetization's motion in the rotating frame for spatial locations with small and large $B_{1}$ amplitudes.

The MR signal equation will depend on the transverse (${\overset{ˆ}{x}}^{\prime }$ and ${\overset{ˆ}{y}}^{\prime }$) components of the magnetization vector $\vec{M}$ as it precesses about ${\vec{B}}_{\mathrm{eff}}$. The magnetization's initial $\hat{z}$-component $M_{z}\hat{z}$ will coincide with ${\vec{B}}_{\mathrm{eff}}$ and will contribute a constant ${\overset{ˆ}{x}}^{\prime }$ component of

(7) $$M_{z}sin(\theta ){\overset{ˆ}{x}}^{\prime }.$$

The magnetization's initial $\overset{ˆ}{x}$ and $\overset{ˆ}{y}$ components will trace a tilted circle in a plane perpendicular to ${\vec{B}}_{\mathrm{eff}}$ as they precess around it. As illustrated in Figure 3 and Supplementary Movie 1, projecting this tilted circle to the transverse plane contributes elliptical ${\overset{ˆ}{x}}^{\prime }$ and ${\overset{ˆ}{y}}^{\prime }$ components, where the ellipse's major axis is along ${\overset{ˆ}{y}}^{\prime }$ and its minor axis is along ${\overset{ˆ}{x}}^{\prime }$, and ellipticity increases with $B_{1}$ via θ. The ellipse contributes a time-varying ${\overset{ˆ}{x}}^{\prime }$ component that adds to $M_{z}$'s ${\overset{ˆ}{x}}^{\prime }$ component to produce a total ${\overset{ˆ}{x}}^{\prime }$ component of:

(8) $${\vec{M}}_{x}^{\prime }\left(t\right)=M_{z}\sin\left(\theta \right){\overset{ˆ}{x}}^{\prime }+\left(M_{x}\cos\left(\omega_{eff}t\right)+M_{y}\sin\left(\omega_{eff}t\right)\right)\cos\left(\theta \right){\overset{ˆ}{x}}^{\prime }.$$

It also contributes a time-varying ${\overset{ˆ}{y}}^{\prime }$ component of:

(9) $${\vec{M}}_{y}^{\prime }(t)=\left(-M_{x}sin\left(\omega_{eff}t\right)+M_{y}cos\left(\omega_{eff}t\right)\right){\overset{ˆ}{y}}^{\prime }.$$

Combining ${\vec{M}}_{x}^{\prime }(t)$ and ${\vec{M}}_{y}^{\prime }(t)$ into a complex-valued rotating frame transverse magnetization $M_{xy}^{\prime }=M_{x}^{\prime }+iM_{y}^{\prime }$ yields:

(10) $$M_{xy}^{\prime }\left(t\right)=M_{z}\sin\left(\theta \right)+\left(M_{x}\cos\left(\omega_{eff}t\right)+M_{y}\sin\left(\omega_{eff}t\right)\right)\cos\left(\theta \right)+i\left(-M_{x}\sin\left(\omega_{eff}t\right)+M_{y}\cos\left(\omega_{eff}t\right)\right).$$

(11) $$=M_{z}\sin\left(\theta \right)+Re\left\{M_{xy}e^{-i\omega_{eff}t}\right\}\cos\left(\theta \right)+iIm\left\{M_{xy}e^{-i\omega_{eff}t}\right\}$$

where $M_{xy}=M_{x}+iM_{y}$ is the complex-valued Larmor frame transverse magnetization.

To obtain an expression for the transverse magnetization detected in the Larmor frame, we shift back to that frame by demodulating $M_{xy}^{\prime }$ as:

(12) $$M_{xy}(t)=M_{xy}^{\prime }e^{i\Delta \omega_{RF}t}$$

(13) $$=\left[M_{z}\sin\left(\theta \right)+Re\left\{M_{xy}e^{-i\omega_{eff}t}\right\}\cos\left(\theta \right)+iIm\left\{M_{xy}e^{-i\omega_{eff}t}\right\}\right]e^{i\Delta \omega_{RF}t}$$

Integrating this expression over space (x) yields the final signal equation:

(14) $$s\left(t\right)=\int \left[M_{z}\left(x\right)\sin\left(\theta \left(x\right)\right)+Re\left\{M_{xy}\left(x\right)e^{-i\omega_{eff}\left(x\right)t}\right\}\cos\left(\theta \left(x\right)\right)+i\;Im\left\{M_{xy}\left(x\right)e^{-i\omega_{eff}\left(x\right)t}\right\}\right]e^{i\Delta \omega_{RF}t}dx,$$

where we have made the spatial dependence of the magnetization, θ, and $\omega_{\mathrm{eff}}$ explicit. The magnetization dynamics during Bloch-Siegert frequency encoding as well as the generated rotating- and Larmor-frame signals are illustrated in Supplementary Movie 1, for two $B_{1}$ amplitudes. A validation of Equation 14 against numerical Bloch simulation is also reported in the Supplementary Information, for the experimental configuration described below.

### Relationship Between RF and $B_{0}$ Frequency Encoding

We now show how the above signal expression relates to $B_{0}$ gradient-based frequency encoding, to illustrate the conditions for which inverse Fourier transform reconstruction is feasible, to facilitate recovery of the transverse magnetization profile via inverse Fourier transform, and to generate intuitive relationships between RF gradient strength, encoded field-of-view, and encoded spatial resolution.

The first step is to note that the unwanted signal component $M_{z}(x)\;sin(\theta (x))e^{-i\Delta \omega_{RF}t}$ will be off-resonance by $\Delta \omega_{RF}$, and if $\Delta \omega_{RF}>\gamma B_{1}$ across the field-of-view (in the present work, $\Delta \omega_{RF}>4\gamma B_{1}$ everywhere in the field-of-view), it can be removed by filtering or cropping the image, so long as the signal is sampled with a frequency of at least $2\Delta \omega_{RF}$. The large size of $\Delta \omega_{RF}$ relative to $\gamma B_{1}$ also enables approximating cos(θ)≈1, leading to a simplified signal equation:

(15) $$s(t)\approx \int \left[Re\left\{M_{xy}(x)e^{-i\omega_{\mathrm{eff}}(x)t}\right\}+i\;Im\left\{M_{xy}(x)e^{-i\omega_{\mathrm{eff}}(x)t}\right\}\right]e^{i\Delta \omega_{RF}t}dx$$

(16) $$=\int M_{xy}\left(x\right)e^{-i\omega_{\mathrm{eff}}\left(x\right)t}e^{i\Delta \omega_{RF}t}dx.$$

Equation (16) is a linear function of a phase-modulated complex-valued transverse magnetization, but it is not yet clear that an inverse Fourier transform may be used to recover $M_{xy}(x)$.

To further equate RF frequency encoding and conventional $B_{0}$ gradient encoding and establish the conditions under which an inverse Fourier transform can be used to reconstruct $M_{xy}(x)$, we note that since $\sqrt{a^{2}+b^{2}}\approx a+\frac{b^{2}}{2a}$ by binomial approximation for a>b, we can approximate:

(17) $$\omega_{eff}=\sqrt{{\left(\Delta \omega_{RF}\right)}^{2}+{\left(\gamma B_{1}\right)}^{2}}$$

(18) $$\approx \Delta \omega_{RF}+\frac{{\left(\gamma B_{1}\right)}^{2}}{2\Delta \omega_{RF}},$$

and substitute this approximation into the signal equation, yielding:

(19) $$s\left(t\right)\approx \int M_{xy}\left(x\right)e^{-i\frac{{\left(\gamma B_{1}\left(x\right)\right)}^{2}}{2\Delta \omega_{RF}}t}dx.$$

Finally, if $B_{1}(x)$ is a square-root field and can hence be written as $B_{1}(x)=\sqrt{cx}$ for some constant c, then we can write the signal equation in the same form as for $B_{0}$ gradient encoding, as:

(20) $$s(t)\approx \int M_{xy}(x)e^{-i\frac{\gamma^{2}c}{2\Delta \omega_{RF}}t}dx$$

(21) $$=\int M_{xy}\left(x\right)e^{-i\gamma Gxt}dx,$$

where

(22) $$G=\frac{\gamma c}{2\Delta \omega_{RF}}$$

is the equivalent $B_{0}$ gradient strength in units of, e.g., millitesla per meter.

Equations (21) and (22) enable the derivation of useful relationships between spatial resolution, encoded field-of-view, RF gradient strength, off-resonance frequency, and encoding time. For a receiver dwell time of $\Delta_{t}$ seconds, the encoded field-of-view (FOV) is given by:

(23) $$FOV=\frac{2\pi }{\gamma G\Delta_{t}}=\frac{4\pi \Delta \omega_{RF}}{\gamma^{2}c\Delta_{t}},$$

which increases with the encoding pulse's frequency offset, and decreases with the RF gradient strength via c. For a total encoding time of T seconds, the encoded spatial resolution $\Delta_{x}$ is given by:

(24) $$\Delta_{x}=\frac{1}{\gamma GT}=\frac{2\Delta \omega_{RF}}{\gamma^{2}cT},$$

so that spatial resolution becomes finer with increasing RF gradient strength (via c) and coarser with increasing encoding pulse frequency offset.

To summarize, the exact signal equation for Bloch-Siegert RF gradient frequency encoding differs from the equation for $B_{0}$ gradient frequency encoding by the presence of an $M_{z}$ signal component and a signal ellipticity that increases with θ. However, if $\Delta \omega_{RF}$ is much larger than $\gamma B_{1}$ across the field-of-view then the z-component can be filtered away and the ellipticity is negligible, yielding a Fourier transform relationship between transverse magnetization $M_{xy}(x)$ and signal s(t). If $B_{1}(x)$ further has a square-root dependence on x, then RF frequency encoding is equivalent to linear $B_{0}$ gradient encoding. As shown in the Supplementary Information, the size of the z-component also decreases as the excitation flip angle approaches 90°. The acquired field-of-view and spatial resolution can be controlled via the encoding pulse frequency offset, the RF gradient strength, the receiver dwell time, and the duration of the frequency encoded readout.

### Simultaneous Transmit and Receive for RF Frequency Encoding

A key challenge hindering RF frequency encoding has been isolating the strong RF encoding signal from the much weaker proton signal. In the present 47.5 mT imaging configuration, the RF encoding pulse transmitted simultaneously during acquisition is 12 orders of magnitude larger than the signal, which will saturate the receiver and can cause irreversible hardware damage. Increasing the frequency separation between the RF encoding pulse and the proton signal could mitigate this, but the efficiency of BS-based RF gradient encoding decreases as the encoding frequency increases. For example, a pulse 10 kHz away from the proton resonance (within a typical receiver bandwidth) induces a 100 times-larger frequency shift than a pulse 1 MHz away (outside a typical receiver bandwidth).

The requirement to use nearby transmit and receive frequencies for RF frequency encoding necessitates a simultaneous transmit and receive (STAR) approach that allows the RF encoding pulse to remain within the detector coil and receiver bandwidth. However, conventional STAR systems are unsuitable and require complex hardware and transmitting at frequencies far from (> 100 kHz) the receiver frequency^39,40^, or they are limited to low-power RF^41^.

**Figure 4:**
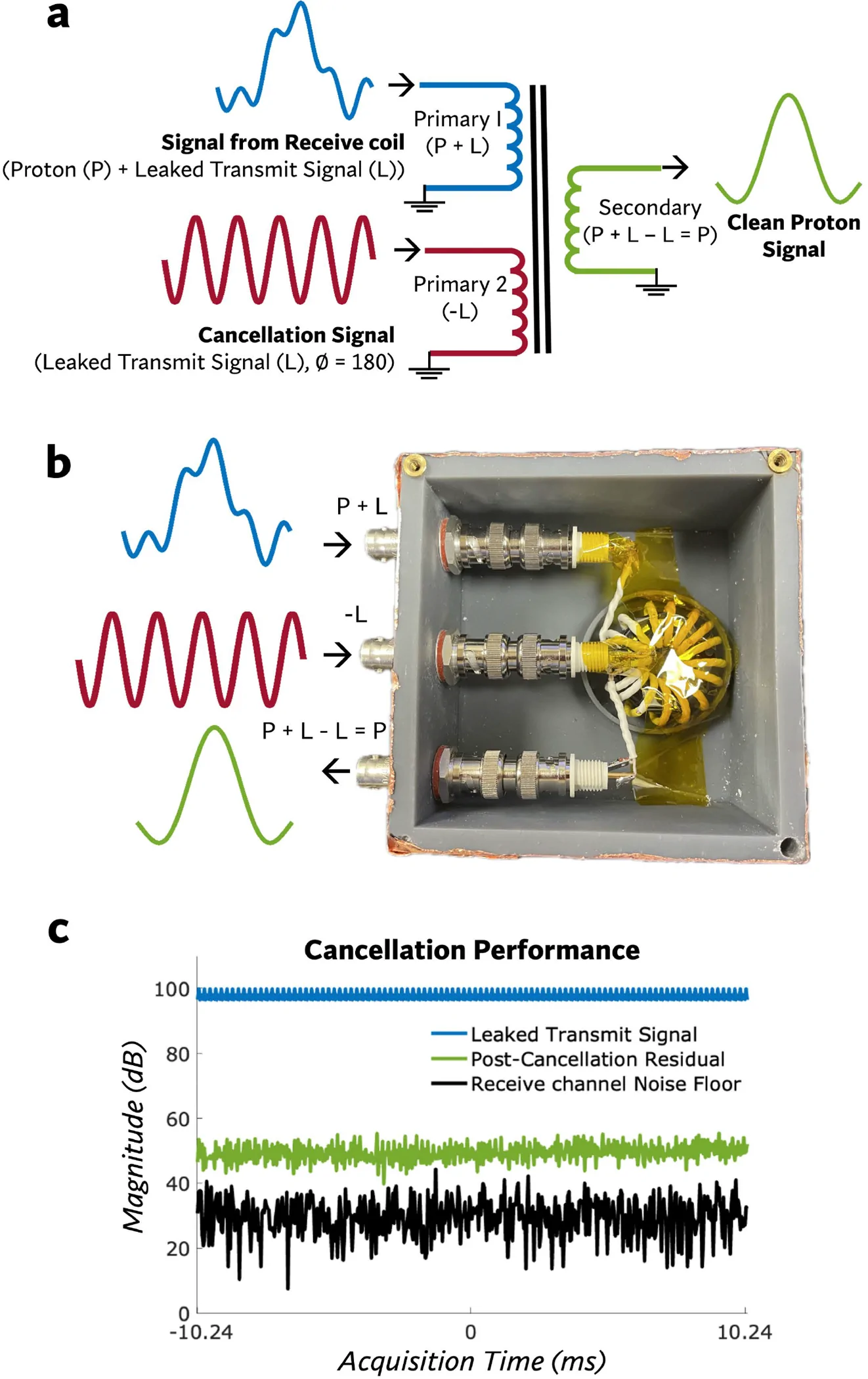
Injection transformer for simultaneous transmit and receive in RF frequency encoding. a) Schematic of the transformer. The signal from the MR receiver coil contains both desired proton signal (P) and unwanted/leaked frequency encoding pulse signal (L). The amplitude and phase of the cancellation signal (-L) are adjusted to cancel the unwanted frequency encoding pulse signal and produce a clean proton signal (P) at the transformer’s secondary winding. b) Constructed injection transformer with 16 tri-windings using Litz wire and a 3.5 cm outer diameter core (T130-14, Micrometals, CA, USA). c) Cancellation performance of the injection transformer during the signal readout, comparing unsuppressed leaked transmit signal from the RF encoding coil, the post-cancellation residual signal using the transformer, and the receive channel noise floor.

To enable RF frequency encoding, we developed a novel injection transformer-based STAR system for simultaneous transmission of a high-power RF encoding pulse at 2.065 MHz while receiving proton signal at 2.075 MHz (the Larmor frequency of protons in our MRI system), which provides high isolation between the transmitted pulse and received signal. Our STAR system (Figure 4a) utilizes a transformer with two primary windings and one secondary that are wound around a core. One primary winding carries the receive coil’s signal, including both the leaked encoding signal from the high-power frequency encoding pulse (L) and the desired but much weaker proton signal (P). The other primary winding receives a cancellation signal comprising only the leaked encoding signal with an inverted phase (-L). Because the secondary winding’s signal will be the sum of the two primary windings’ signals, this configuration produces a clean proton signal on the secondary winding (P + L − L = P), achieving isolation before amplification.

While transformer fundamentals are straightforward, practical implementation requires careful design to achieve the desired voltage conversion. Core and copper losses are the primary factors affecting transformer efficiency. To minimize copper losses, the constructed transformer as shown in Figure 4b employed Litz wire, which has low AC resistance. To reduce core losses, a powdered iron core (T-130-30, Micrometals, CA, USA) was used. Validation of this setup was performed using a suitable console, RF encoding, and receive coil configuration. Figure 4c shows that the injection transformer achieved 99.66% encoding signal cancellation during active RF frequency encoding. The insertion loss was measured separately using a vector network analyzer (Supplementary Figure 2) and was −1.42 dB at the Larmor frequency (2.075 MHz), corresponding to 71.6% power transmission. Vector network analyzer measurements of the injection transformer (Supplementary Figure 2) confirmed that the insertion loss remains flat across ±100 kHz from the Larmor frequency, demonstrating frequency-independent performance across the bandwidth relevant for RF frequency encoding. This design including components and formers had a total cost less than $200.

### Validation of concept

**Figure 5:**
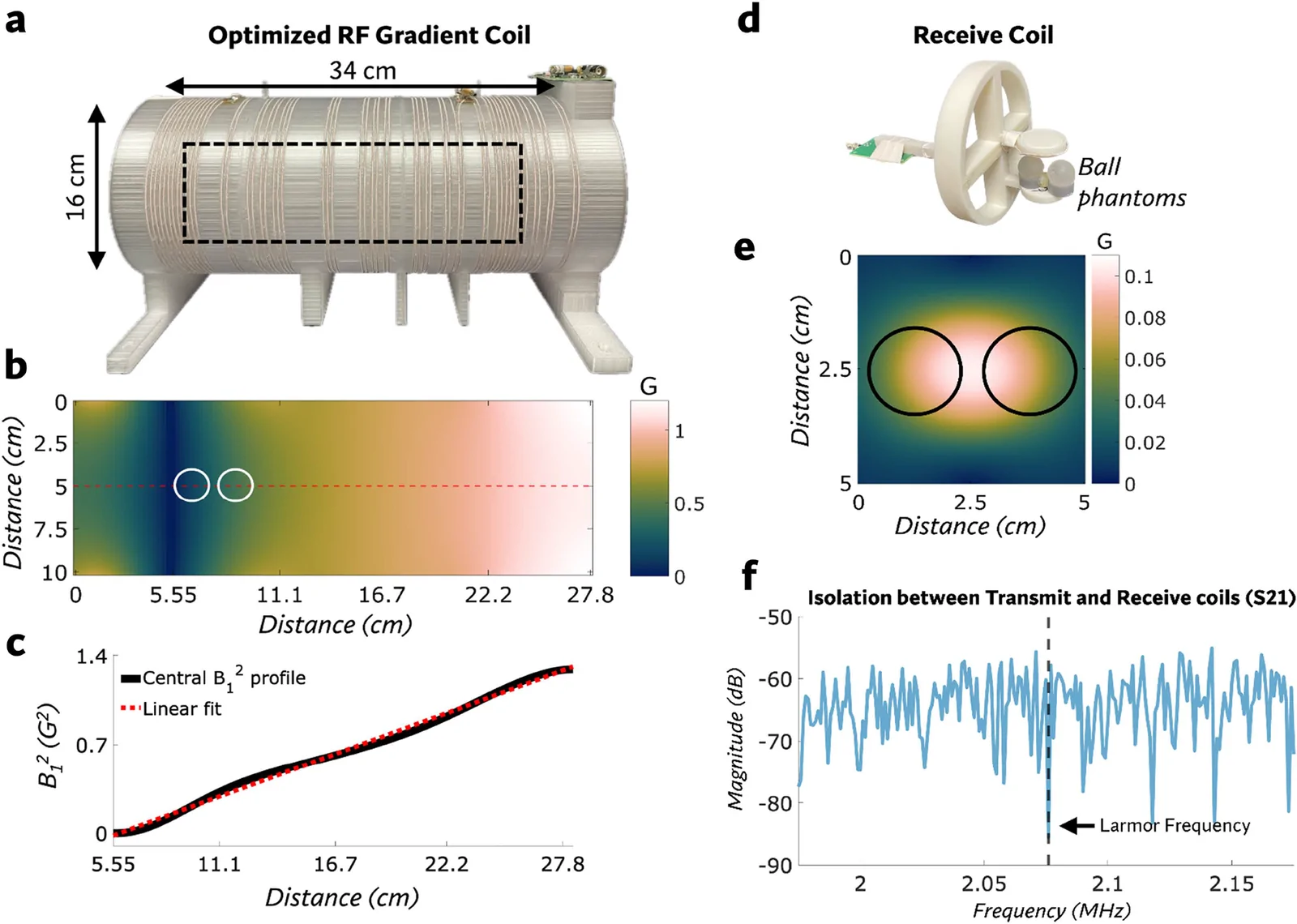
RF coils for RF frequency-encoding. a) Constructed optimized RF gradient solenoidal coil used for transmitting both frequency encoding and imaging excitation pulses. b) Simulated $B_{1}$ (radiofrequency field) map along the central profile of the coil, with the color bar indicating $B_{1}$ amplitude in Gauss (G), and the locations of the two ball phantoms indicated by white circles. c) $B_{1}$^2^ (squared radiofrequency field) profile along central profile in the $B_{1}$ map indicated by the dashed red line in the usable FOV. d) Constructed receive surface coil with two 2 cm ball phantoms. e) Receive coil sensitivity, with the color bar indicating $B_{1}$ amplitude in Gauss (G), and the locations of the two ball phantoms indicated. f) An S_21_ (forward transmission coefficient) measurement between the RF gradient transmit coil and the receive coil shows the high isolation between them.

To validate RF frequency encoding, an optimized solenoidal RF gradient coil was constructed (Figure 5a). Because the BS frequency shift is proportional to the square of the $B_{1}$ field amplitude, the coil was optimized to produce a square-root-shaped $B_{1}$ field^29^. To achieve a true $B_{1}$ null at one end of the field of view, the coil incorporated counter-wound bucking windings. The optimized coil had a total of 52 windings, 12 of which were counter-wound bucking windings. Figure 5a also shows the coil’s simulated central $B_{1}$ map and its mean $B_{1}$^2^ profile with an R-squared value of 0.9974. Experimental $B_{1}$ mapping^29,42^ confirmed the accuracy of the field profile, with an R-squared value of 0.9981. This coil had a −3dB BW of 40 kHz and was used to transmit both the excitation and encoding pulses in the GRE pulse sequence.

Figure 5b shows the single-turn 3.5 cm-diameter surface coil that was used to receive signals from two 2 cm-diameter spherical phantoms in the GRE pulse sequence. This coil was geometrically decoupled from the gradient transmit coil (Figure 5c; > 60 dB decoupling at the Larmor frequency) to reduce the amplitude of the transmitted RF encoding pulses prior to cancelling it with the injection transformer. The phantoms experienced a $B_{1}$ gradient between 0 and 0.55 G within a 4.5 cm-long region indicated in the $B_{1}$ map of Figure 5a.

**Figure 6:**
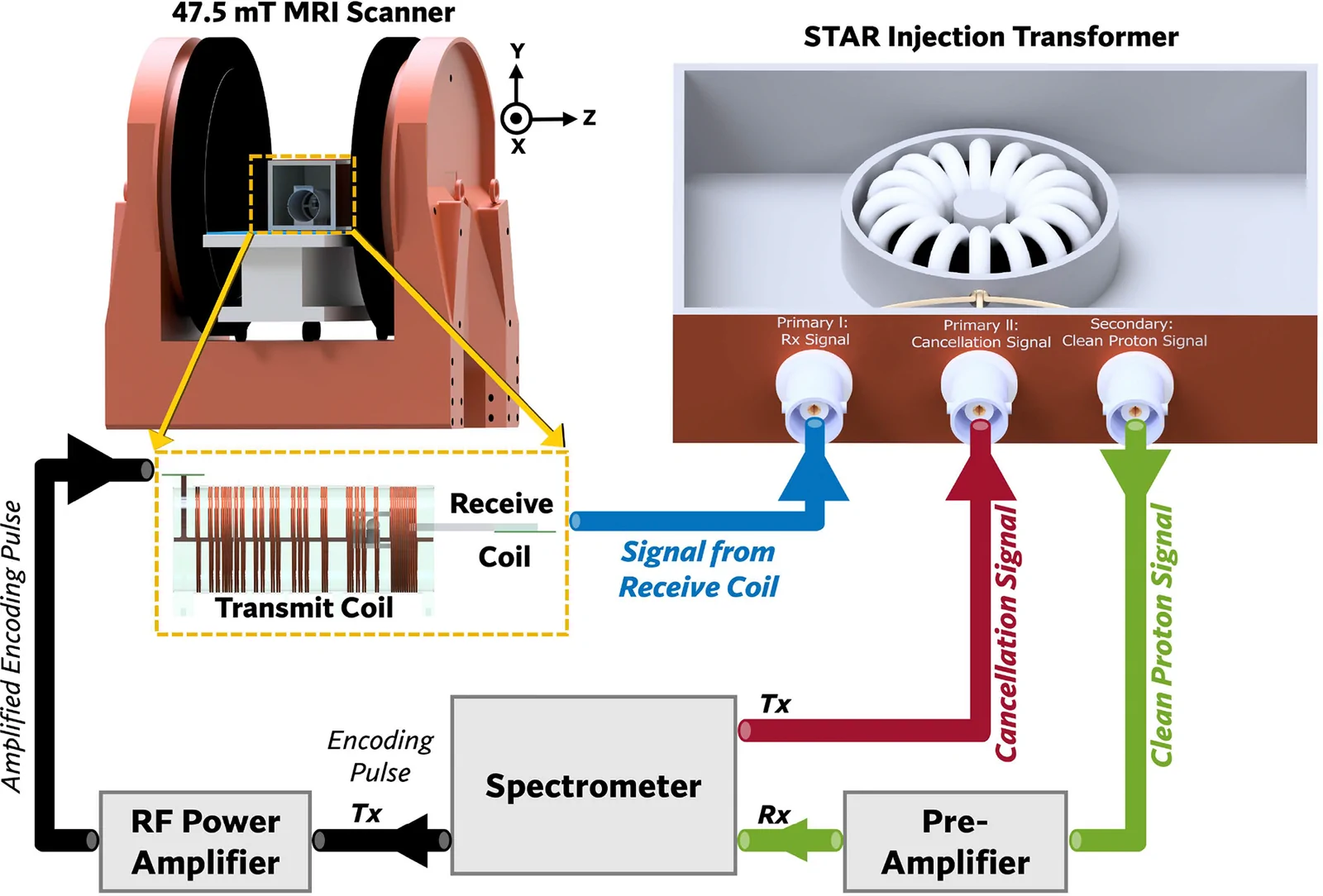
Experimental setup with a 47.5 mT open MRI scanner. The spectrometer generated encoding, imaging, and cancellation pulses, and received signal. Encoding and imaging pulses were amplified by the RF power amplifier and transmitted to the RF gradient coil. RF coils were housed within an RF shield. The received signal passed through the Simultaneous Transmit and Receive (STAR) injection transformer, and the clean proton signal was amplified before it was recorded by the spectrometer. Blue, red, and green arrows represent the received signal, cancellation signal, and clean proton signal, respectively.

The overall experimental setup is illustrated in Figure 6. The coils and phantoms were placed on the bed of a 47.5 mT open MRI scanner with an RF shield to minimize external interference. A Tecmag spectrometer console (Redstone, Tecmag, Houston, USA) executed the GRE sequence including all gradient pulses (not illustrated in Figure 5), all RF pulses, and all signal reception. RF excitation and encoding pulses were amplified by a Tomco 2 kW amplifier (BT02000-AlphaA-100ms, Tomco Technologies, Stephney, AUS) which was connected to the RF gradient coil. Signals from the receive coil were input to one primary winding of the STAR injection transformer, and the spectrometer generated the cancellation signal that was input to the other primary winding. The transformer’s secondary winding carried the clean proton signal which was input to a low-noise pre-amplifier (MITEQ P/N 158310057, NY, USA) and then recorded by the spectrometer’s receiver. While a powerful 2 kW power amplifier was used for transmission, the peak power used by the frequency encoding RF pulses throughout all experiments was 12 W.

**Figure 7:**
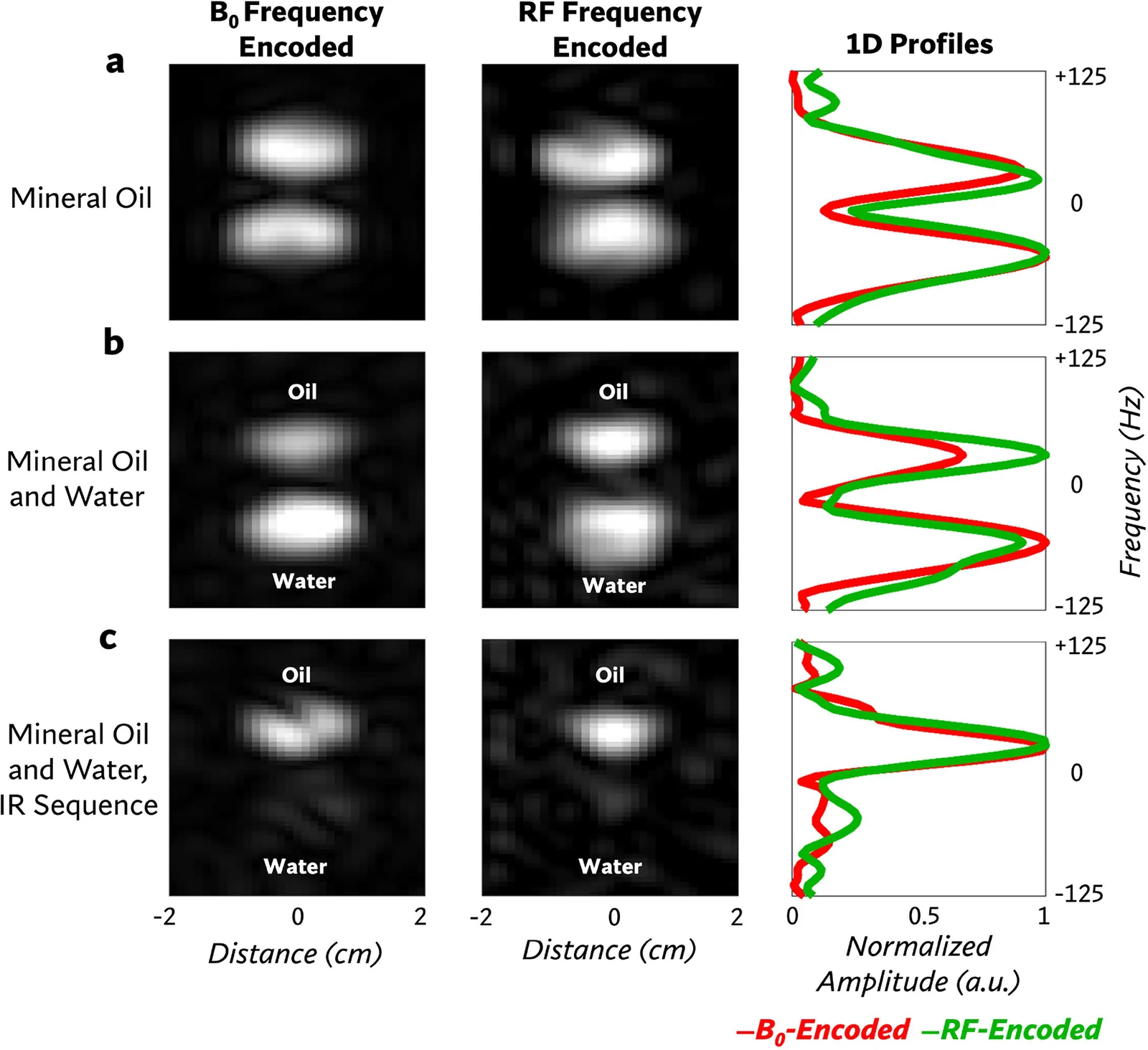
RF frequency-encoded images. a) Images of two mineral oil-filled ball phantoms acquired with $B_{0}$ gradient frequency encoding (left) and RF frequency encoding (right), using a 2D gradient-recalled echo (GRE) sequence where the vertical image dimension is frequency-encoded, and the horizontal axis is $B_{0}$ (main magnetic field) gradient phase-encoded. b) Images of water- and mineral oil-filled ball phantoms acquired with $B_{0}$ gradient (left) and RF frequency encoding (right), using a 2D GRE sequence. c) Image of water and mineral oil filled ball phantoms acquired with $B_{0}$ gradients (left) and RF frequency encoding with a 2D inversion recovery GRE sequence (right). One-dimensional profiles along the center of the phantom in the vertical frequency encoding direction are shown on the right for each case.

Figure 7 shows images generated by the RF frequency encoding experiments using the GRE sequence of Figure 2b with parameters: readout bandwidth = 25 kHz, number of readout points = 512, number of phase-encodes = 34, readout duration = 20.48 ms, acquisition time per average = 15.19 s, BS encoding pulse offset frequency = 10 kHz, TE = 36.7 ms, TR = 447 ms, number of averages = 300. The images were reconstructed by inverse Fourier transform, which was facilitated by a small maximum tilt angle θ=13.2° given the 10 kHz frequency offset and maximum $B_{1}$ of 0.55 G, yielding a minimum cos(θ)=0.974 across the field-of-view. The asymmetric sampling matrix (512 frequency-encode points × 34 phase-encode steps) was chosen for two reasons. First, acquiring many points along the frequency-encoding dimension is inexpensive in terms of scan time, and the correspondingly large FOV along that dimension ensures that the off-resonant M_z_ signal component, which is displaced by Δω_RF_ from the image center, does not alias into the reconstructed image. Second, using only 34 phase-encode steps reduced the total acquisition time per average to 15.19 seconds while providing adequate resolution to separate the two phantoms along the phase-encode dimension.

Figure 7a shows images of two ball phantoms filled with mineral oil, acquired using the 2D GRE pulse sequence with $B_{0}$ and RF frequency encoding, as well as 1D profiles of each along the frequency-encoded (vertical) image dimension. The third spatial dimension was unencoded in all images, which were all reconstructed by inverse 2D Fourier transform. The reconstructed images had 3.9 mm spatial resolution in the frequency-encoded dimension, and the effective gradient strength was 1.32 G/m. The images agree in terms of geometric dimensions and contrast of the balls.

To demonstrate image contrast preservation with RF frequency encoding, the GRE scans were first repeated using phantoms filled with water and mineral oil (Figure 7b) with TE = 29.7 ms, TR = 1.24 s and image acquisition time per average = 42.16 s. The $B_{0}$ and RF frequency-encoded images again matched well. The experiment was then repeated with an Inversion Recovery (IR) sequence wherein an inversion pulse played 61 ms before the excitation pulse, in order to impart *T*_1_ weighting that should reduce signal from the ball containing water. The images resulting from this sequence are shown in Figure 7c, which do match in terms of attenuation of the water ball signal. Phase images corresponding to the magnitude images in Figure 7 are shown in Supplementary Figure 7.

## Discussion

This work demonstrated the feasibility of RF frequency-encoded MRI by addressing two fundamental challenges: the mechanistic challenge of applying a frequency gradient to transverse magnetization without significant out-of-plane rotations, and the hardware challenge of isolating weak MR signals from the strong RF encoding signals during simultaneous transmission and reception at nearby frequencies. By developing a Bloch-Siegert-based frequency encoding pulse sequence in conjunction with an injection transformer-based STAR system and an optimized RF gradient coil with a square-root-shaped $B_{1}$ field, we were able to generate RF frequency-encoded MR images which were concordant with $B_{0}$ frequency-encoded images in both structure and contrast.

Our results have many implications for the future of MRI. RF gradient-based frequency encoding offers several advantages over conventional $B_{0}$ gradient encoding: reduced hardware complexity, lower cost, improved patient comfort through silent operation, reduced power consumption enabling more sustainable design, and design flexibility unconstrained by main field orientation. The technique is particularly attractive for low-field MRI systems, where $B_{0}$ gradient design, performance, and cost present greater challenges, potentially enabling widespread deployment in point-of-care and resource-limited settings. Finally, RF frequency encoding provides an additional degree of freedom in the development of more compact, affordable, and accessible MRI scanners, since it frees the system designer from generating an encoding coil whose field aligns with the scanner’s main field. RF frequency encoding gradient systems need not comprise a single coil but could be implemented as coil arrays that can flexibly adapt the shape of the gradient field within a pulse sequence or for different imaging targets. Supplementary Figure 1 shows an example of an RF coil array^43^ with 24 elements that achieves 3% linearity (squared $B_{1}$ fields) over a target volume in two dimensions.

For this proof-of-concept demonstration, a single-turn receive coil was chosen to maximize geometric decoupling from the transmit coil (> 60 dB isolation) and to provide a wide −3 dB bandwidth (132 kHz) that uniformly covers the 25 kHz readout bandwidth. Future implementations will employ multi-turn receive coils combined with optimized injection transformer designs to achieve the higher SNR necessary for clinical applications.

The conservative RF power level of 12 W used in this work was not a fundamental constraint but reflected three practical considerations. First, higher $B_{1}$ amplitudes increase the magnetization tilt angle θ, reducing the detected signal amplitude proportional to cos(θ). Second, the constant-frequency readout pulse, which was chosen to simplify STAR cancellation, causes unwanted excitation at higher power levels, as illustrated in Supplementary Figure 9 for the flat versus swept prephasor pulse cases. Third, higher transmitted power increases the amplitude of the leaked signal, placing greater demands on the injection transformer's cancellation performance. The RF power requirement also depends on the encoding frequency offset $\Delta \omega_{RF}$: since the Bloch-Siegert frequency shift scales as $B_{1}^{2}/\Delta \omega_{RF}$, lower offsets require less power for a given gradient strength but operating too close to resonance compromises STAR isolation. The 10 kHz offset used here was chosen to balance encoding efficiency with practical isolation requirements and represents a design trade-off that can be optimized for different system configurations. Future implementations using swept-frequency readout pulses, improved transformer isolation, and active coil decoupling will enable higher RF power and faster acquisitions while maintaining image quality.

Supplementary Figure 2 shows the injection transformer-based STAR system introduced here incurs a −1.42 dB insertion loss, which is comparable to passive components such as filters and circulators used in other STAR and self-interference cancellation (SIC) systems^39^. Future implementations could reduce insertion loss through improved transformer core materials and winding configurations.

Cancellation calibration in the current implementation is performed manually by iteratively adjusting the amplitude and phase of the cancellation signal while monitoring the residual leaked signal, taking approximately 5 minutes per coil configuration. Because the high geometric isolation between coils (> 60 dB) minimizes load-dependent coupling variations, calibration does not need to be repeated between scans or with different phantom loads, and only requires repetition if coil positions or cable routing change. Automated calibration algorithms for the injection transformer could reduce setup time to less than 1 minute, comparable to routine MRI calibrations such as center frequency determination or flip angle optimization. The calibration would consist of: (1) transmitting the encoding pulse without sample excitation, (2) measuring the residual signal at the transformer output, and (3) iteratively adjusting the cancellation signal amplitude and phase to minimize the residual below the receiver dynamic range. This process is readily implemented in scanner console software and would make the technique suitable for clinical workflows.

While the RF gradient coil’s $B_{1}$ variation across the imaged ball phantoms was not so great as to prevent clear visualization of the phantoms, for clinical translation, a separate uniform-field excitation coil would be preferable to avoid flip angle inhomogeneity across the object, with the RF gradient coil dedicated solely to spatial encoding. Future work will extend the technique to 3D imaging by combining with well-established RF-based slice selection^35,36^ and RF-based phase encoding^29,38^ techniques, evaluate in vivo performance, and assess its potential for clinical applications, particularly in point-of-care and resource-limited settings.

While further advancements are needed in areas such as Bloch-Siegert pulse optimization, active coil decoupling, and digital filtering, our work establishes the crucial foundation for RF-based frequency encoding. We anticipate a trajectory of innovation paralleling conventional $B_{0}$ gradient development, where dramatic improvements in gradient fidelity and strength have fundamentally expanded MRI capabilities. RF frequency encoding opens a new frontier for making MRI technology globally accessible through compact, affordable, and silent systems that can transform medical imaging in diverse clinical environments.

## Methods

### Injection Transformer Design and Construction

To build an efficient injection transformer by minimizing core losses, a toroidal core made of carbonyl iron powder material (T130-14, Micro metals, TN, USA) was employed. This core has an outer diameter of 35 mm and inductance of 14 nH and exhibits favorable characteristics for higher frequencies due to its lower flux density and reduced eddy current losses, owing to its “powdered” material composition. To minimize copper losses, Litz wire was utilized instead of single-strand wire. A drill was used in the winding process to achieve even braiding of the Litz wire, which consisted of multiple flexible strands. Heat shrink material was used to secure the windings in place. The transformer configuration incorporated a total of 16 tri-windings. The optimized injection transformer was securely housed in a 3D-printed enclosure that featured three feedthroughs for external connections. Copper tape was used to shield the box. Efficiency calculations were conducted to assess the performance of this setup, employing a network analyzer for characterization.

### RF Gradient Encoding and Receive Coil Design and Construction

The RF gradient transmit coil was designed for wrist/hand imaging with a square-root $B_{1}$ field profile^29^. For this proof-of-concept demonstration with small phantoms positioned in the central region of the coil, the coil's large size provided uniform encoding gradients while maximizing geometric decoupling (> 60 dB) from the smaller receive coil, which was chosen to maximize receive sensitivity.

The RF gradient encoding coil was designed using an extension of the integer linear programming-based method^29,44^ to produce the square-root field required to generate a linear frequency gradient with the Bloch-Siegert shift. The method involves determining the number of integer coil windings in a set of predefined locations along the length of the solenoid to produce a target field pattern. The coil’s length was 34 cm and its diameter was 16 cm, in order to provide sufficient geometric isolation between the transmit and receive coils for the proof of principal experiment. The optimization was performed in MATLAB (Mathworks, Natick, MA, USA) using the intlinprog() function. It began by defining a set of coil grooves (M = 30) along the length of the solenoid which were distributed with square-root pitch. For each groove position m, a single loop coil's $B_{x}$ field was calculated at a set of points (N=58) uniformly distributed on the x-axis ${\left\{B_{l,m}\left(x_{n},0,0\right)\right\}}_{n=1}^{N}$. Square-root $B_{1}$ fields were set as the target field values at the same x-axis locations ${\left\{B_{t}\left(x_{n},0,0\right)\right\}}_{n=1}^{N}$, and the number of windings at each coil groove $I_{m}\left(0\leq I_{m}\leq 4\right)$ was determined to satisfy:

$$\frac{\sum_{m=1}^{M}B_{l,m}\left(x_{n}\right)I_{m}\;-\;B_{t,n}\left(x_{n}\right)}{B_{t,n}\left(x_{n}\right)}\leq \delta$$

which was enforced at each of the x-axis locations ${\left\{\left(x_{n},0,0\right)\right\}}_{n=1}^{N}$.

$B_{1}$ field maps were calculated using the Biot-Savart law in MATLAB with 1 mm spatial resolution. This quasi-static approximation is valid at our operating frequency (2.075 MHz, wavelength ≈145m) where the coil dimensions (34 cm length, 16 cm diameter) are much smaller than the wavelength (≪λ). While the coil optimization can generate a coil with a square-root field pattern, the coil's ${B_{1}}^{+}$ field cannot begin at zero since the ${B_{1}}^{+}$ field of even a single RF loop falls off exponentially and the optimization could not compensate for this, as it did not have the ability to switch current directions in the loops. To increase the numerically optimized coil's linearity, its field intercept was nulled by adding bucking windings (counter-wound loops) to one end. This addition was done manually in simulation by adjusting the number of opposite-current turns until the slope of the ${\left({B_{1}}^{+}\right)}^{2}$ field was maximized while its amplitude was zero on one end of the coil. This resulted in addition of 12 bucking windings for a total of 52 windings.

Based on the results of the above optimization and bucking winding addition, to minimize the likelihood of arcing between windings and due to that coil's higher inductance, its grooves (30) containing multiple windings were separated out to multiple grooves (52) with one winding each centered at the original groove position, with a 1 mm gap between the grooves. The final coil was then designed. Three distributed 75 pF tuning capacitors were added to the coil for peak power distribution^18,45^. The coil was constructed using Fused Deposition Modeling (makexyz, AZ, USA) based 3D-printed formers, where the former without the windings were first designed and the winding patterns were subtracted from the main former in CAD (Autodesk inventor) to produce grooves for the windings. Litz wire was used for RF coil construction due to its low AC resistance. The inductances of the coils were measured at 20 kHz using an LCR meter, and their S-parameters were measured using a vector network analyzer (Keysight N9925A-Fieldfox, Santa Rosa, CA, USA). The RF gradient encoding coil’s inductance was 164 uH, its Q was 51.8, and its −3 dB BW was 40 kHz. This coil was used both for transmitting the encoding and the excitation pulses.

The 3.5 cm diameter surface receiver RF coil former shown in Figure 4B was designed to fit snugly within the RF gradient encoding coil. The coil former was Stereolithography (Formlabs 3, Boston, MA) 3D-printed. The coil former had an extrusion on which the two 2 cm (outer diameter) ball phantoms were placed with a gap of 0.5 cm between them. This occupied a 4.5 cm region along the x-axis in the RF gradient coil with a 0–0.55 G $B_{1}$ range. The Receive coil’s Q was 15.7 and its −3 dB BW was 132 kHz. The low-field MRI scanner used in this study employs a coordinate system where the main magnetic field $B_{0}$ (z) is oriented left-to-right across the patient, while the x-direction corresponds to the head-to-foot axis as shown in Figure 6. This represents a rotated coordinate system compared to conventional clinical MRI scanners, where $B_{0}$ typically points head-to-foot and the z-direction is along the patient bore. The RF receive coil was placed polarized along the scanner’s y direction. The RF gradient encoding coil was placed polarized along the scanner’s *x* direction. This provided high decoupling between the two as shown in Figure 5c. Supplementary Figure 3 shows the Sparameters of both coils and the S_21_ measurement.

### Experimental setup

The overall positioning of the coils in a 47.5 mT scanner along with a portable RF shield is shown in Figure 6. The RF shield was constructed using press fitted T-slot aluminum framing and FR4 boards with 2 oz copper with the following dimensions: 24 in (Length) × 12 in (Width) × 12 in (Height). It was fully enclosed in the front after inserting the coil for maximum shielding. The transformer as described previously was used for active signal cancellation. The first primary winding was connected to the RF imaging receive coil. The second primary winding was connected to the input port of the cancellation signal played through a Tecmag spectrometer (Tecmag, Houston, TX) and an active TR switch^46^ to prevent any reflection of power into the spectrometer. The secondary winding was connected to the receive channel in the spectrometer through a low-noise preamplifier with 70 dB gain (MITEQ P/N 1583 10057, NY, USA). The cancellation amplitude and phase were calibrated by iteratively adjusting the cancellation signal while monitoring the residual leaked signal at the transformer's secondary winding, ensuring it remained below the receiver's dynamic range (achieving > 45 dB cancellation). For our coil configuration with high geometric isolation (> 60 dB), this calibration remained stable across different phantom loads and multiple scans. Recalibration was only required when coil positions or cable routing changed.

### Pulse Sequence Design

A 2D GRE sequence as shown in Supplementary Figure 4 was used to implement RF frequency encoding. RF frequency encoding was applied along the length of the RF gradient encoding/transmit coil (x) and $B_{0}$ gradient based phase encoding was applied along the coil’s diameter (y). A non-selective excitation pulse was used and thus the signal was projected across the remaining dimension (z). As shown in Supplementary Figure 5a, a 20.48 ms long constant FM/AM pulse was used as the RF frequency encoding readout pulse (K_BS_ = 116.57 rad/G^2^, $\Delta \omega_{RF}=-10\;kHz$). A 12.91 ms long ’U’-shaped frequency swept off-resonant pulse shown in Supplementary Figure 5b was used as the RF frequency encoding pre-phasor pulse (K_BS_ = 65.95 rad/G^2^, $\Delta \omega_{RF}=10\;kHz$). The swept pulse was designed so that most of the BS shift phase accrual occurs during the flat, smaller-off resonance part of the pulse’s FM waveform, and the sweeps at the ends serve to minimize on-resonance excitation^35^. A peak RF power of 12 W was used for the entire image encoding acquisition.

The 2D GRE sequence collected 34 $B_{0}$ phase encodes (G_y_ = 0.694 mT/m) and 512 read out points (readout acquisition time = 20.48 ms) with an imaging bandwidth of 25 kHz and 300 averages. The sequence has a TE= 36.7 ms, TR = 447 ms, image acquisition time per average = 15.19 s for the experiment with the mineral oil balls (Fig 7a) and TE = 29.7 ms, TR = 1.24 s, image acquisition time per average = 42.16 s for the experiments that used both mineral oil and water (Fig 7b–c). The inversion recovery sequence used a T_i_ = 61 ms, image acquisition time per average = 44.23 s (Fig 7c). The sequence also included a calibration scan which has the same imaging sequence but without the excitation pulse interleaved. This calibration scan was included in case digital filtering was required to remove post-cancellation residual signal in post-processing.

Prior to imaging, we calibrated the cancellation pulse for the STAR transformer for amplitude and phase. The leaked transmit signal was shaped as shown in Supplementary Figure 6a. To reshape this signal, a pre-emphasized RF frequency encoding pulse was used, resulting in a smaller variation as shown in Supplementary Figure 6a. The post-cancellation residual signal was further reduced by 50% when compared to the post-cancellation residual signal with an uncompensated BS encoding pulse as shown in Supplementary Figure 6b. This also ensured that the protons experience a flat encoding signal and avoids any additional excitation during encoding.

### Image Reconstruction and Validation

In order to reconstruct the RF frequency-encoded images, the first 19 points were cropped in the time domain to eliminate any transients arising from both the encoding and the cancellation pulses, bringing the total number of readout points to 493. The signal was then windowed using a Tukey filter (tukeywin() function in MATLAB) with a weight of 0.4. The k-space data were zero-padded, extending the x-axis by 1080 points on each side and the y-axis by 42 points on each side. The image was then inverse FFT-reconstructed resulting in the images shown in Figure 7. The effective gradient strength was calculated using Equation (22), and the nominal acquired spatial resolutions was calculated using Equation (24).

As shown in Figure 7, $B_{0}$ frequency-encoded image was acquired using gradient strengths matched to that of the RF frequency encoding experiment for comparison and was cropped, windowed and zero-padded similarly for an inverse Fourier transform reconstruction. While digital filtering was not used in the reconstructions shown in Figure 7, Supplementary Figure 8 shows its utility. Supplementary Figure 8 shows the cropped, windowed and zero-padded inverse Fourier transform reconstruction for the $B_{0}$ frequency-encoded and the RF frequency-encoded experiments. For the RF frequency encoding experiments, results of the sequence with excitation and without excitation (calibration scan) are shown. Subtracting the two, a difference image is shown with the post-cancellation residual leakage signal removed. Supplementary Figure 9 shows the effect of using a constant AM/FM pre-phasor with the same image processing steps described earlier.

## Supplementary Material

Reviews

Gradient Coil Array

Caption

Encoding Video

## Data Availability

The data that support the findings of this study are openly available at https://github.com/grissomlab/Gradient_Free_Frequency_Encoded_MRI

## Code Availability

Custom code for Bloch simulation and visualization of results is openly available at https://github.com/grissomlab/Gradient_Free_Frequency_Encoded_MRI
