# Supplementary material for: Frequency-Encoded Magnetic Resonance Imaging with Dynamic Radio Frequency Field Gradients
Source: Commun Phys. Author manuscript; Available in PMC 2026 Aug 20. (PMC13488602; doi:10.1038/s42005-026-02686-5)
Supplement: Supplementary file 1 [file NIHMS2200620-supplement-Caption.pdf]

Description of Additional Supplementary Files

File name- Supplementary Movie 1

File description- Magnetization trajectory (left) and generated rotating- and Larmor-frame signals (right), for a 30-degree excitation pulse and a 20.48 ms-long RF frequency encoded readout with a 10 kHz encoding pulse resonance offset and B₁ amplitudes of 0.25 (first part of video) and 2.5 G (second part of video). Only the first 2.5 ms of the 2.5 G signal is shown. The elliptical trajectory traced by the rotating-frame magnetization is plotted in black in the x'-y' plane.
