# Supplementary material for: Frequency-Encoded Magnetic Resonance Imaging with Dynamic Radio Frequency Field Gradients
Source: Commun Phys. Author manuscript; Available in PMC 2026 Aug 20. (PMC13488602; doi:10.1038/s42005-026-02686-5)
Supplement: Supplementary file 2 [file NIHMS2200620-supplement-Gradient_Coil_Array.pdf]

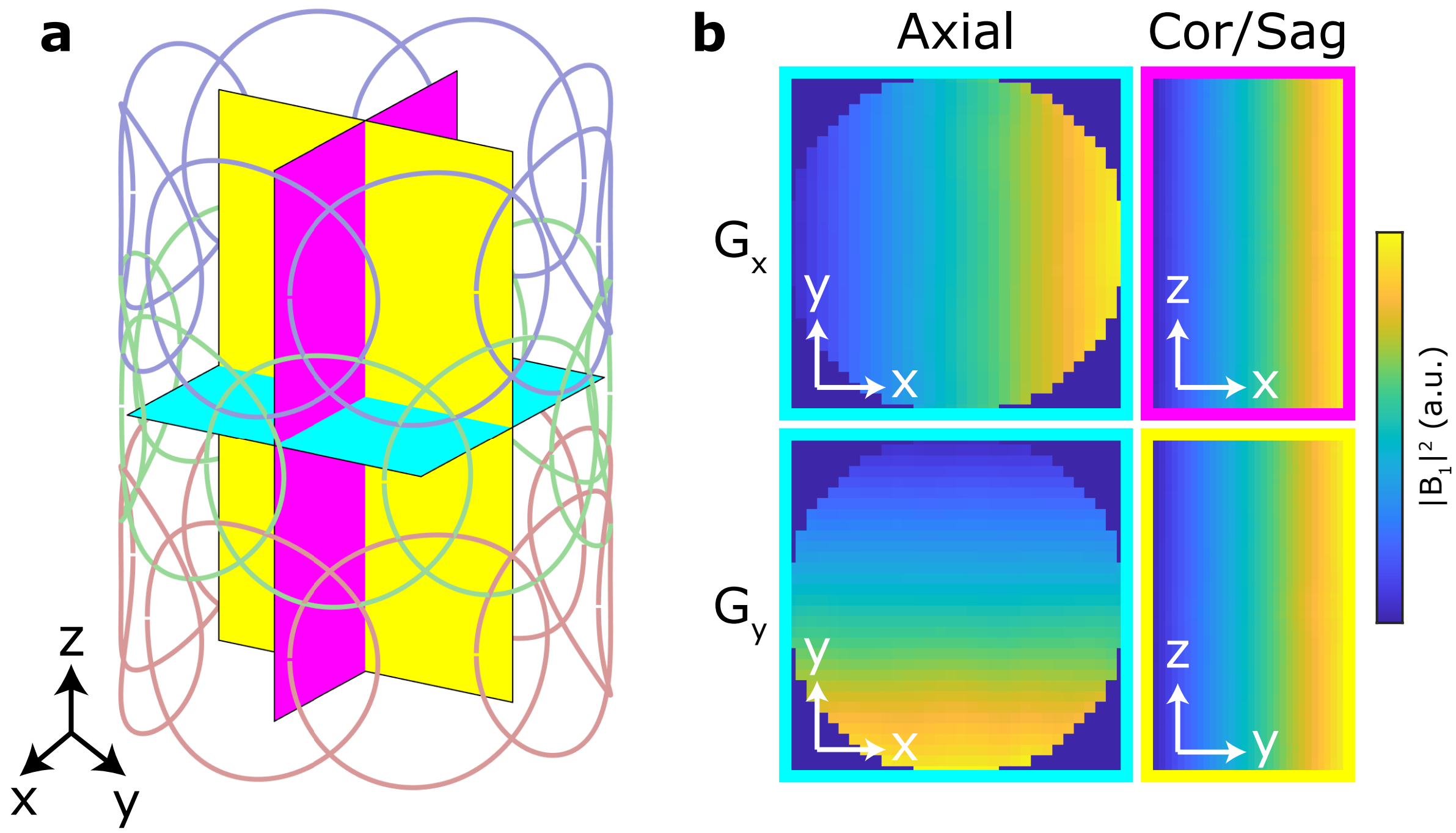

Supplementary Figure 1: *Example of a RF gradient coil array. A) RF gradient coil with 24 elements (3 rows of 8 loops). The array is 54 cm tall and 24 cm in diameter, with 12 cm-diameter overlapped loops. A set of complex-valued RF weights were designed for each axial slice in a 17 cm-diameter cylindrical inner ROI, to produce square-root B_1 field magnitudes. b) The squared magnitudes of the resulting B_1 fields, in the color-coded planes indicated in (a). The squared fields are highly linear, with maximum errors of 3%.*

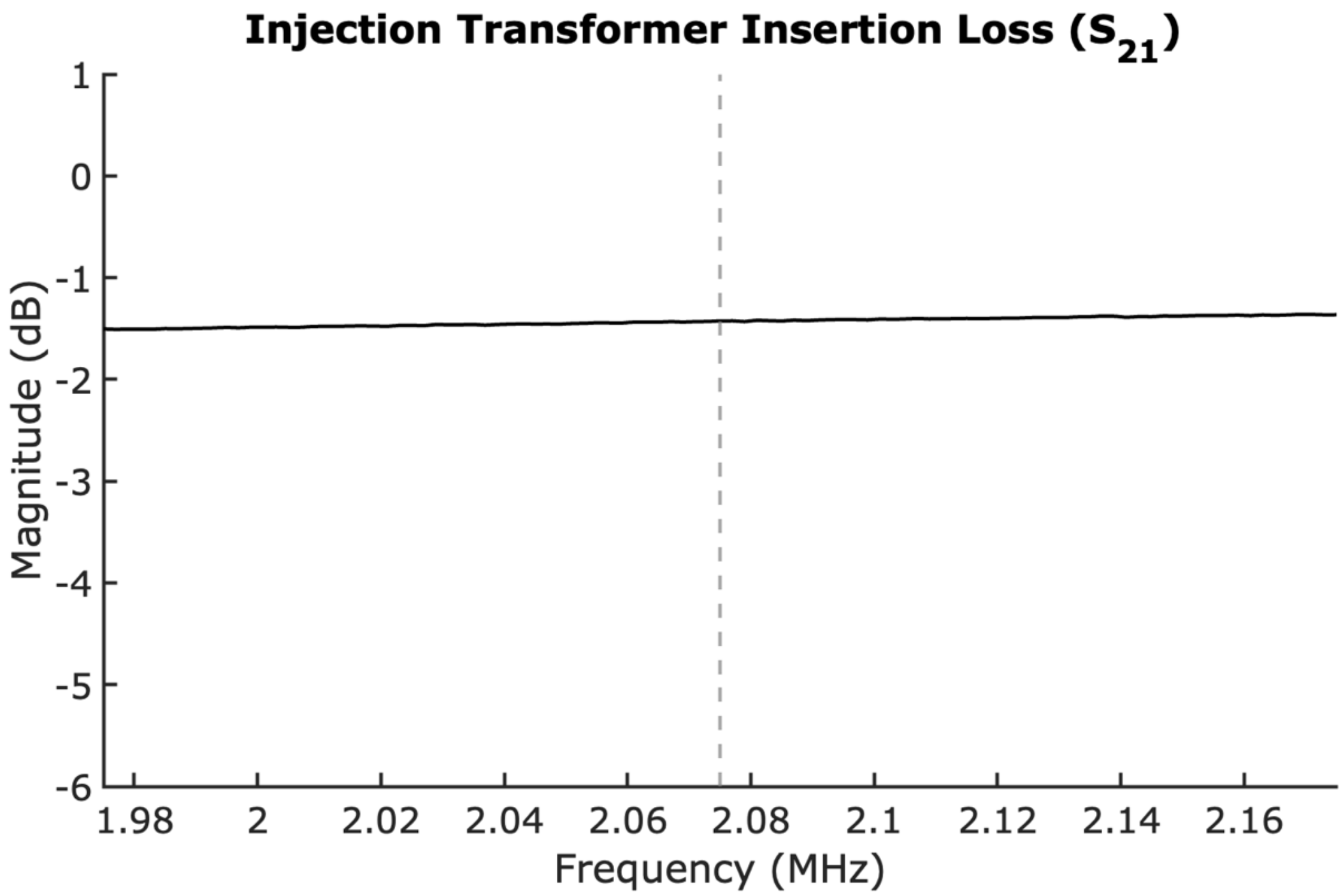

Supplementary Figure 2: Frequency-dependent characterization of the injection transformer using vector network analyzer measurements. S_{21} between primary winding 1 and the secondary measured from 1.975 MHz to 2.175 MHz (±100 kHz around the 2.075 MHz Larmor frequency). The insertion loss remains flat across this bandwidth (between -1.50 and -1.36 dB), confirming frequency-independent transformer performance.

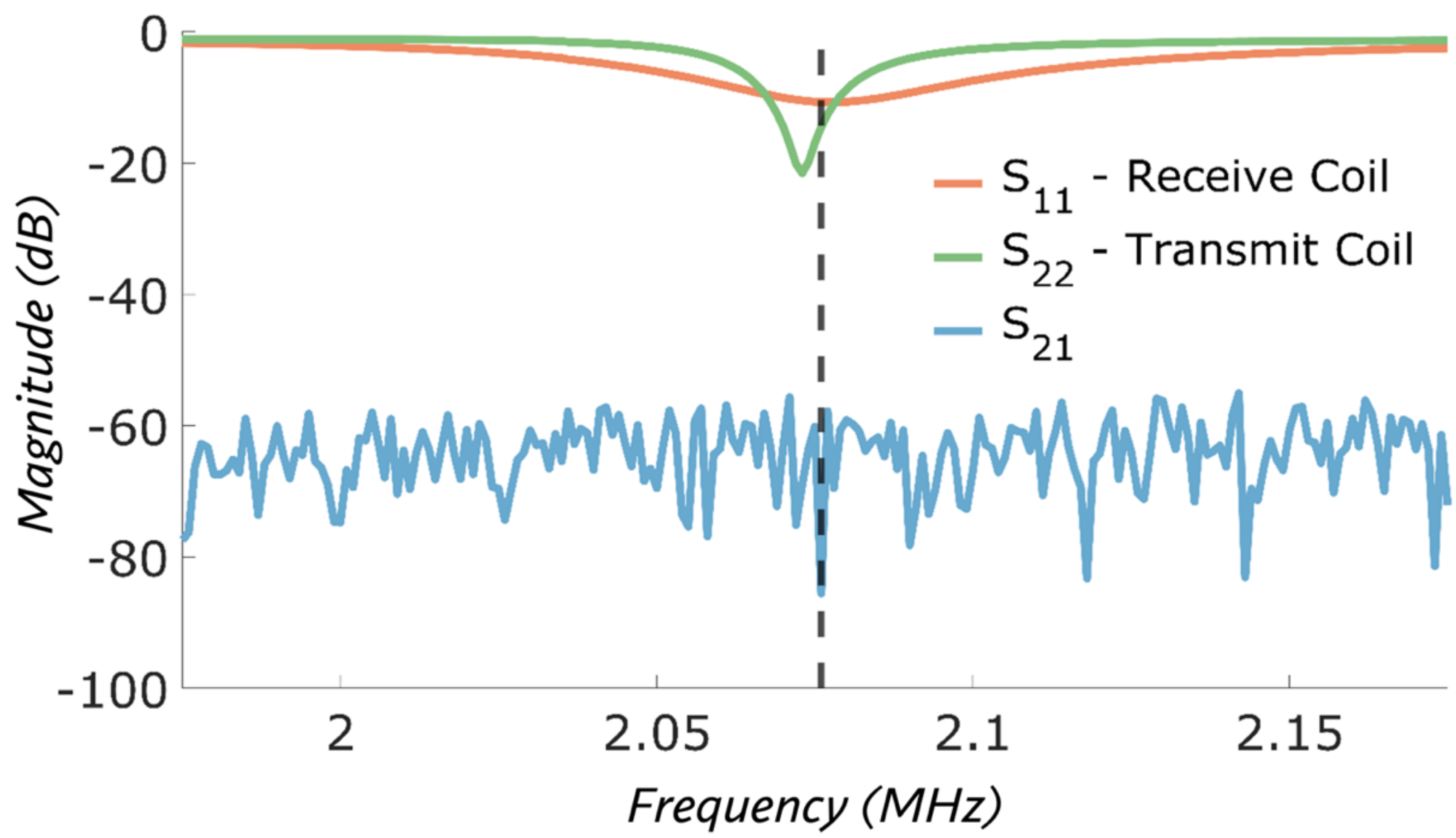

Supplementary Figure 3: S-parameters of the RF gradient coil (Q = 51.8, BW = 40 kHz), the RF receive coil (Q = 15.7, BW = 132 kHz), and their isolation.

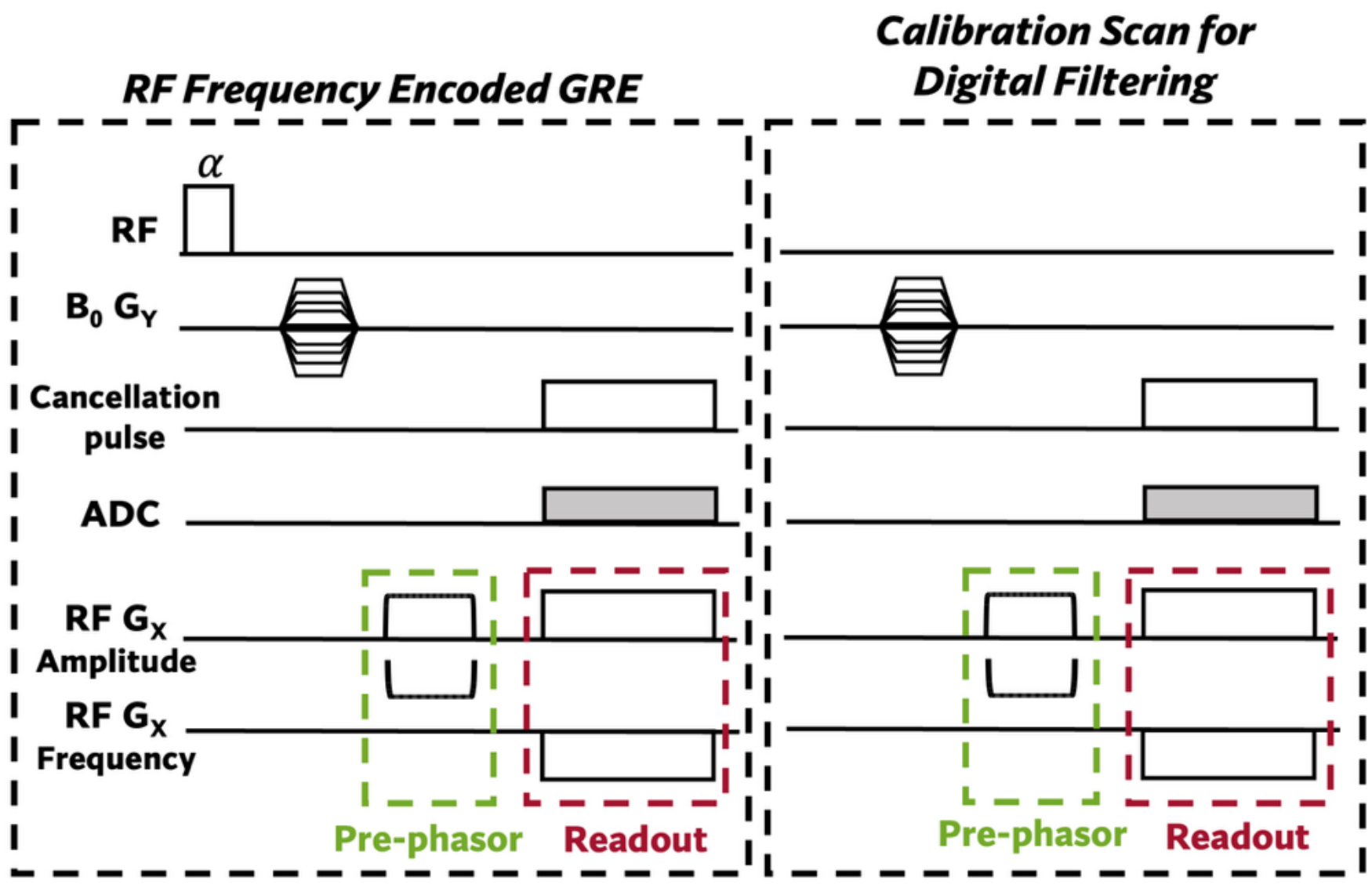

Supplementary Figure 4: *The 2D GRE pulse sequence for RF frequency-encoded imaging. A calibration scan without the excitation pulse was interleaved to remove post-cancellation residual signal as shown in Supplementary Figures 8 and 9.* Detailed pulse waveforms are shown in Supplementary Figure 5.

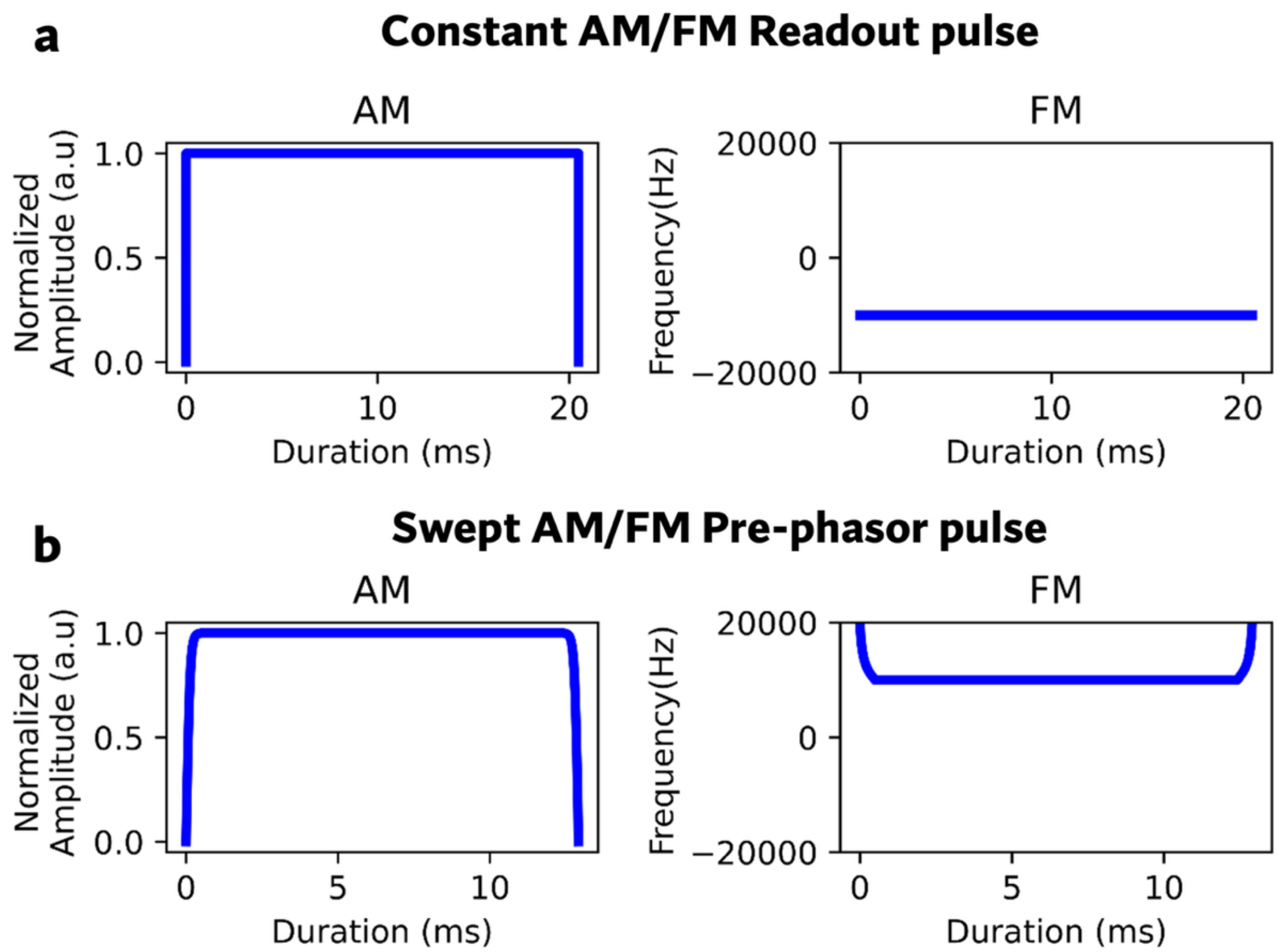

Supplementary Figure 5: *RF frequency encoding pulse waveforms. a) The 20.5 ms-long constant AM/FM Readout pulse's AM and FM waveforms. b) The 12.9 ms-long swept AM/FM pre-phasor pulse's AM and FM waveforms. The FM waveform contains frequency sweeps at the beginning and end that keep the magnetization locked around the effective RF magnetic field prior to and after the smaller-offset central portion, during which most of the phase encoding occurs*[1]*.*

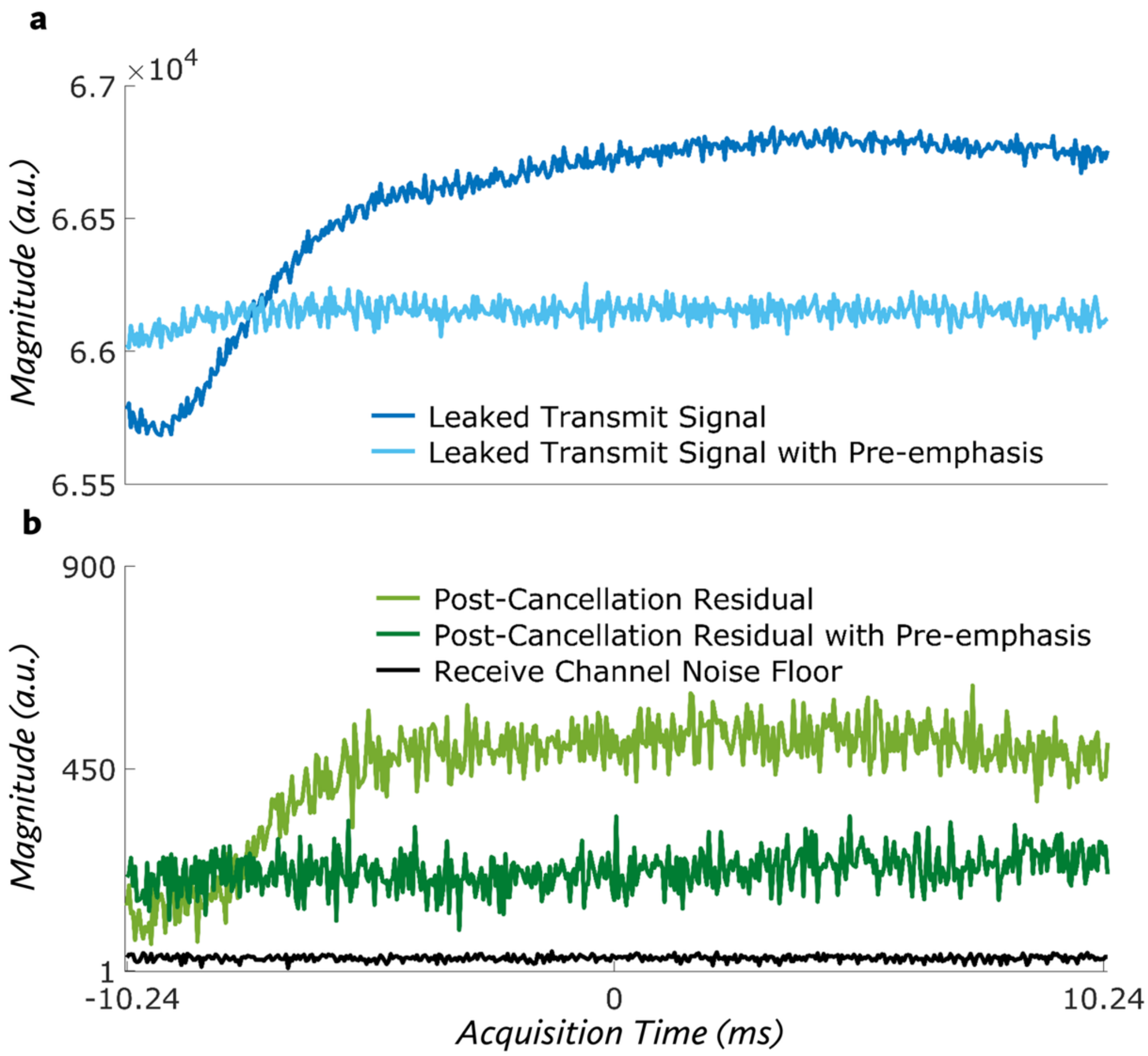

Supplementary Figure 6: *Importance of pre-emphasizing the RF frequency encoding pulse. a) Comparison of flat (uncompensated) and pre-emphasized leaked transmit signals, before active cancellation. b) Comparison of the post-cancellation residual signal using flat and pre-emphasized BS encoding transmit pulses. The receive channel noise floor is shown for reference. Using pre-emphasized encoding pulses results in 50% greater reduction in post-cancellation residual signal when compared with flat encoding pulses.*

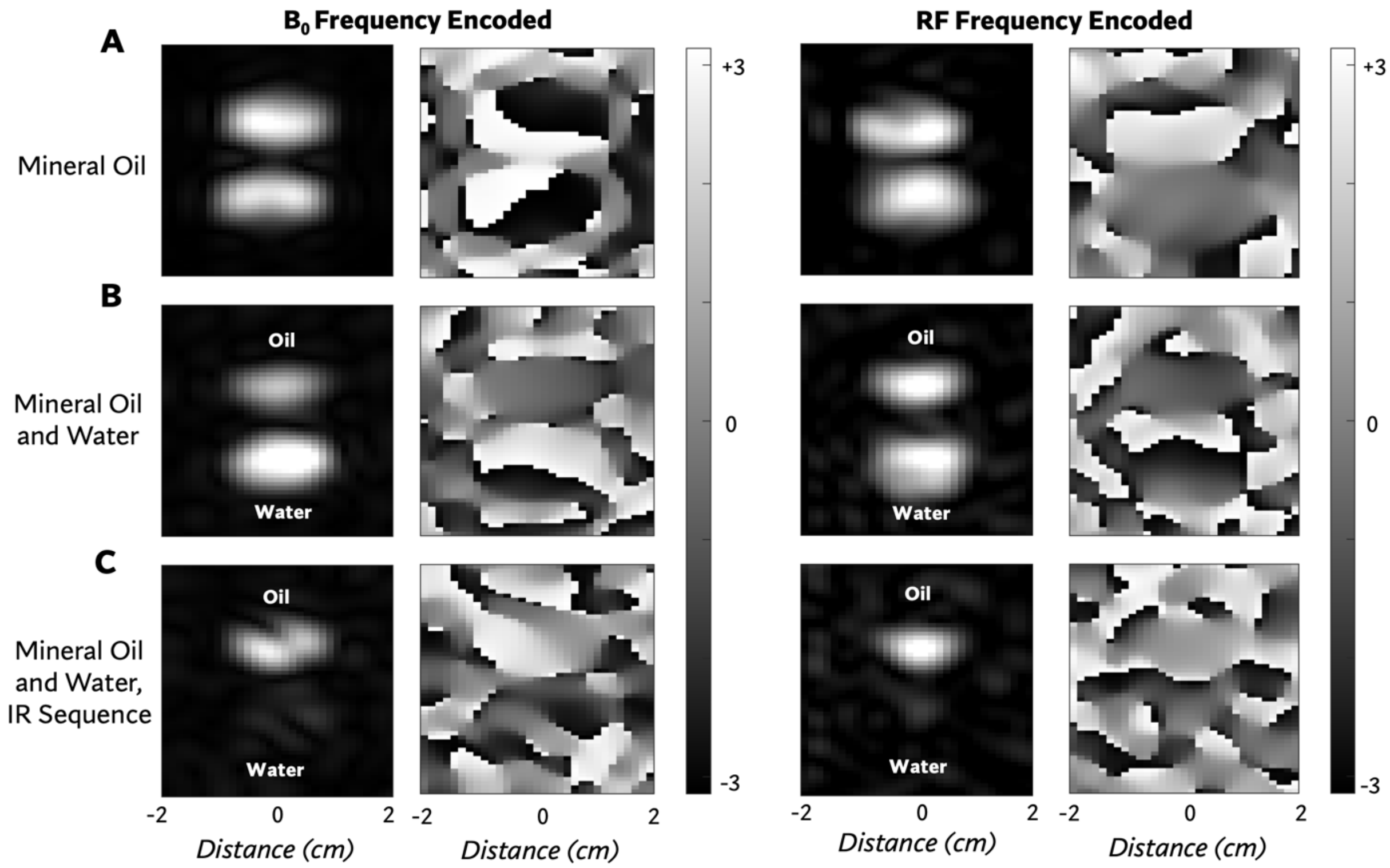

Supplementary Figure 7: Magnitude (left) and phase (right) images for a) two mineral oil-filled ball phantoms, b) water- and mineral oil-filled ball phantoms, and c) inversion recovery (IR) experiments with water and mineral oil phantoms. For each row, B_0 gradient frequency encoding is shown on the left and RF frequency encoding is shown on the right.

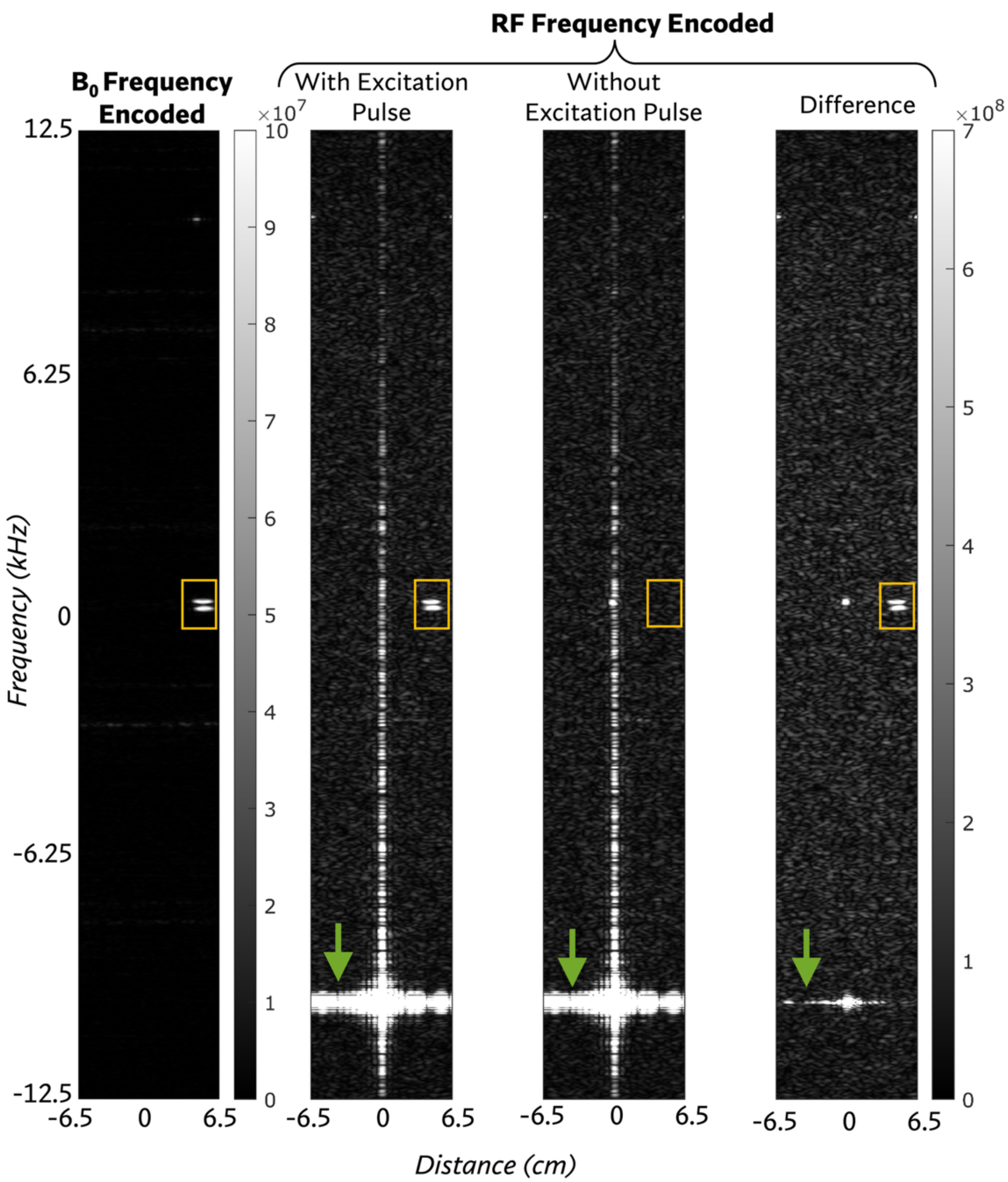

Supplementary Figure 8: *Windowed and zero-padded inverse FFT-reconstructed images, to illustrate all signal components. (Left to Right) A B_0 frequency-encoded image and RF frequency-encoded images with an excitation pulse, without an excitation pulse (the calibration scan) and a difference image to remove the post-cancellation residual signal. This illustrates a potential solution for removing post-cancellation residual signal. The yellow box indicates the location of the two-ball phantom. The green arrows indicate the post-cancellation residual encoding signal.*

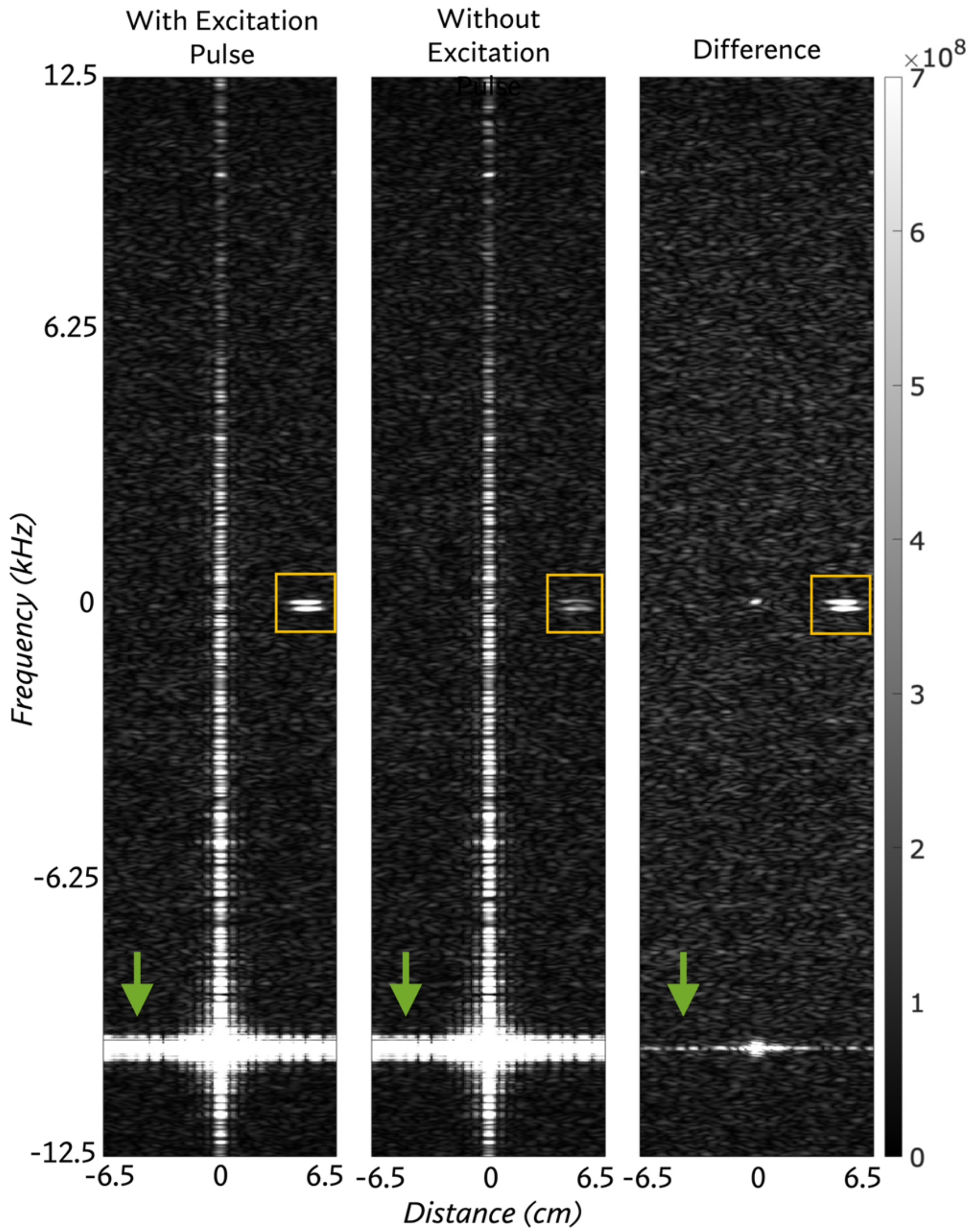

Supplementary Figure 9: *The importance of using swept pre-phasor pulses, illustrated by windowed and zero-padded inverse FFT-reconstructed RF frequency-encoded images (left) with an excitation pulse, (middle) without an excitation pulse (the calibration scan) and (right) their difference to remove the post-cancellation residual signal. The image without an excitation pulse still shows the two-ball phantom due to excitation caused by the constant AM/FM pre-phasor pulse, motivating the use of the swept pulse. The yellow box indicates the location of the two-ball phantom. The green arrows show the post-cancellation residual encoding signal.*

Supplementary Movie 1 (Attached File): *Magnetization trajectory (left) and generated rotating- and Larmor-frame signals (right), for a 30-degree excitation pulse and a 20.48 ms-long RF frequency encoded readout with a 10 kHz encoding pulse resonance offset and* B_1 *amplitudes of 0.25 (first part of video) and 2.5 G (second part of video). Only the first 2.5 ms of the 2.5 G signal is shown. The elliptical trajectory traced by the rotating-frame magnetization is plotted in black in the x′-y′ plane.*

Supplementary Note 1: Signal Equation Validation

To verify the Bloch Siegert frequency encoding signal equation (Equation 14), a simulation was performed with parameters that matched the imaging experiment. Supplementary Figure 10a plots an initial longitudinal magnetization profile corresponding to the two imaged balls, a square-root B_1 field scaled to a maximum of 0.55 G, yielding a maximum θ = 13.2° and a minimum $\cos(\theta)$ = 0.974 across the field-of-view for an off-resonant pulse frequency ($\Delta\omega_{RF}$) of 10 kHz. Supplementary Figure 10b plots Bloch-simulated signal versus signal predicted by Equation 14, which match closely, including the highly oscillatory 10 kHz component corresponding to the $M_z(x)\sin(\theta(x))\ e^{i\Delta\omega_{RF}t}$ term.

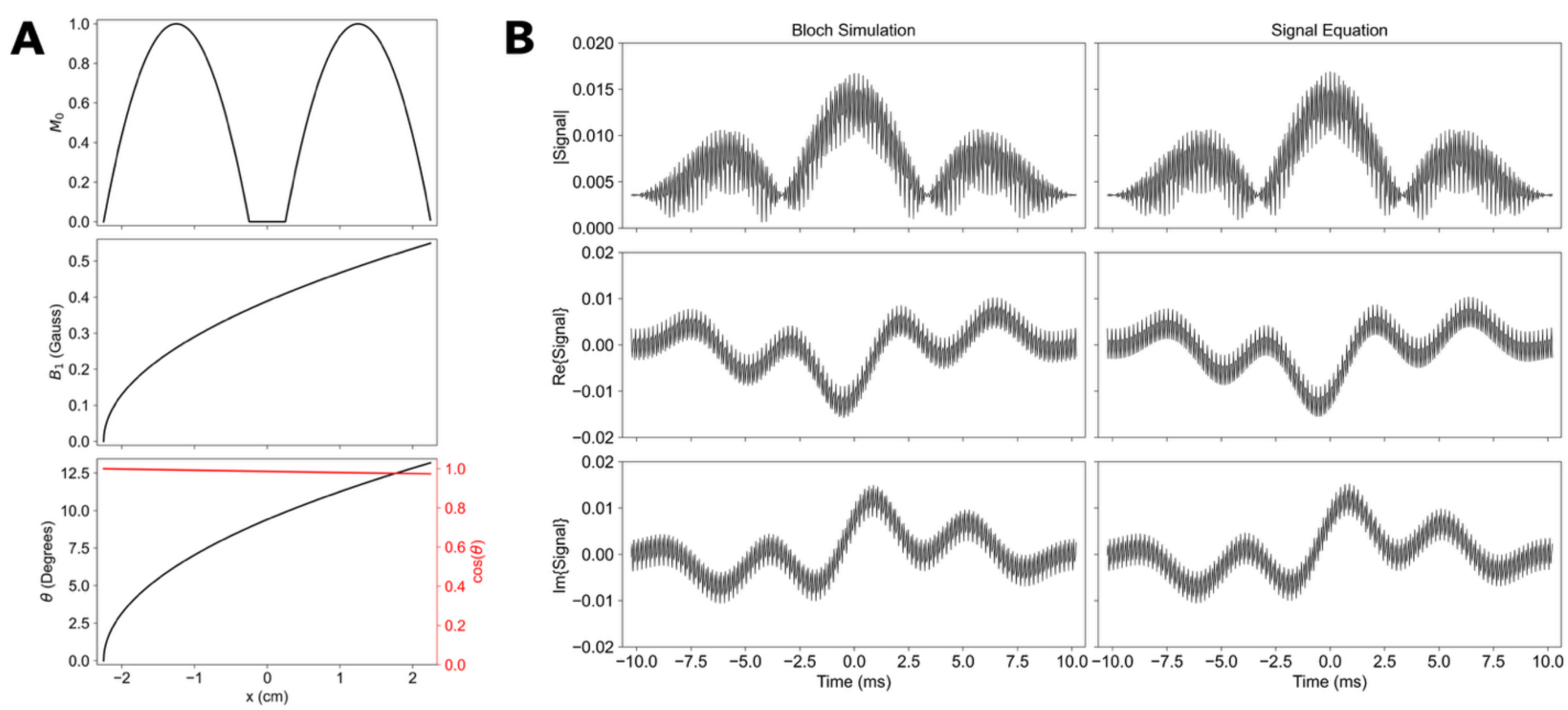

Supplementary Figure 10: *a) RF Frequency encoding simulation setup. Top: Magnetization (M_0) profile of the two balls used in the experiment, with the same diameters and spacing. Middle: Square-root B_1 profile, scaled to the same peak of 0.55 G, as used in the experiment. Bottom: Tilt angle θ and cos (θ) ≈ 1 across the object. b) Bloch-simulated signal (left) versus the signal predicted by Equation 14, for the two-ball object. The signals match, and both contain the expected 10 kHz-off-resonant $M_z(x)$ component.*

z-Component Filtering

Supplementary Figure 11a plots the spectrum of the Bloch-simulated signal before and after filtering and shows that the unwanted signal component is easily suppressed without affecting the lower frequency primary signals. Supplementary Figure 11b plots the real and imaginary components of the Bloch-simulated signal post-filtering, which no longer contains the high-frequency modulation.

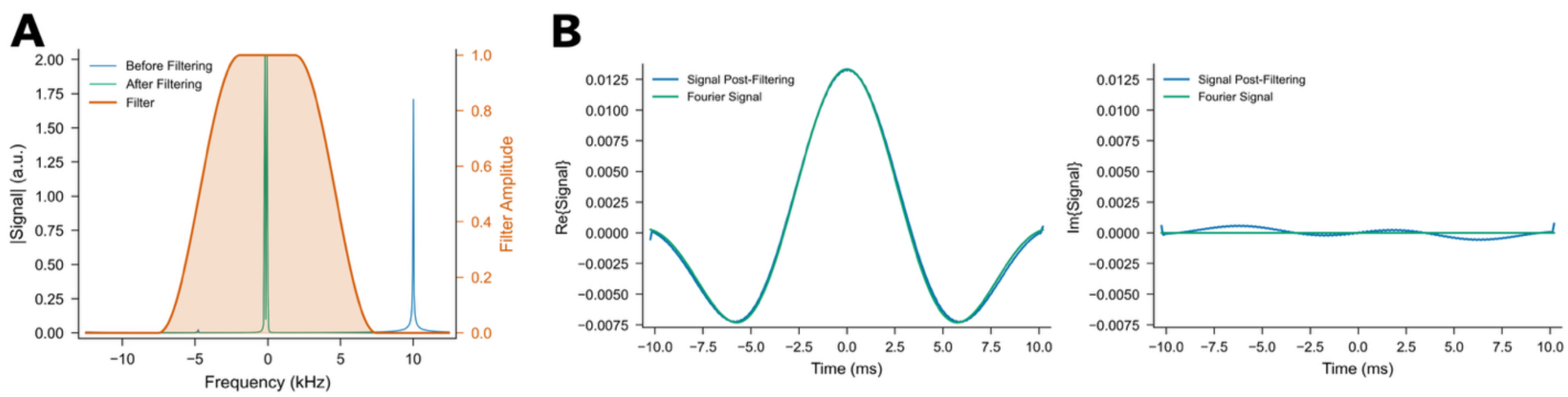

Supplementary Figure 11: *a) Spectrum of the RF frequency-encoded signal before (blue) and after (green) filtering. The two middle spikes in the signal correspond to the two balls, but there is an unwanted* M_z *signal at the* $\Delta\omega_{RF}$ *frequency (10 kHz), which is removed by the Tukey filter, without affecting the desired signals. b) Real (left) and imaginary (right) Bloch-simulated signal components post-filtering (blue), versus signal components predicted by the Fourier signal equation (Equation 21).*

Inverse Fourier Transform Reconstruction and Dependence of Resolution on RF Gradient Amplitude

Supplementary Figure 12a plots an inverse Fourier transform-reconstructed profiles from the simulated two-ball phantom, where the simulated signal matched the experimental settings with $\frac{\Delta\omega_{RF}}{2\pi}$ = 10 kHz encoding pulse offset and c = 6.72 Gauss^2/meter. The two balls are visible in the profile with no appreciable artifacts. Supplementary Figure 12b shows that if the

B_1 amplitude is doubled (corresponding to a 4x increase in *c* and *g*), then finer spatial resolution is achieved as expected.

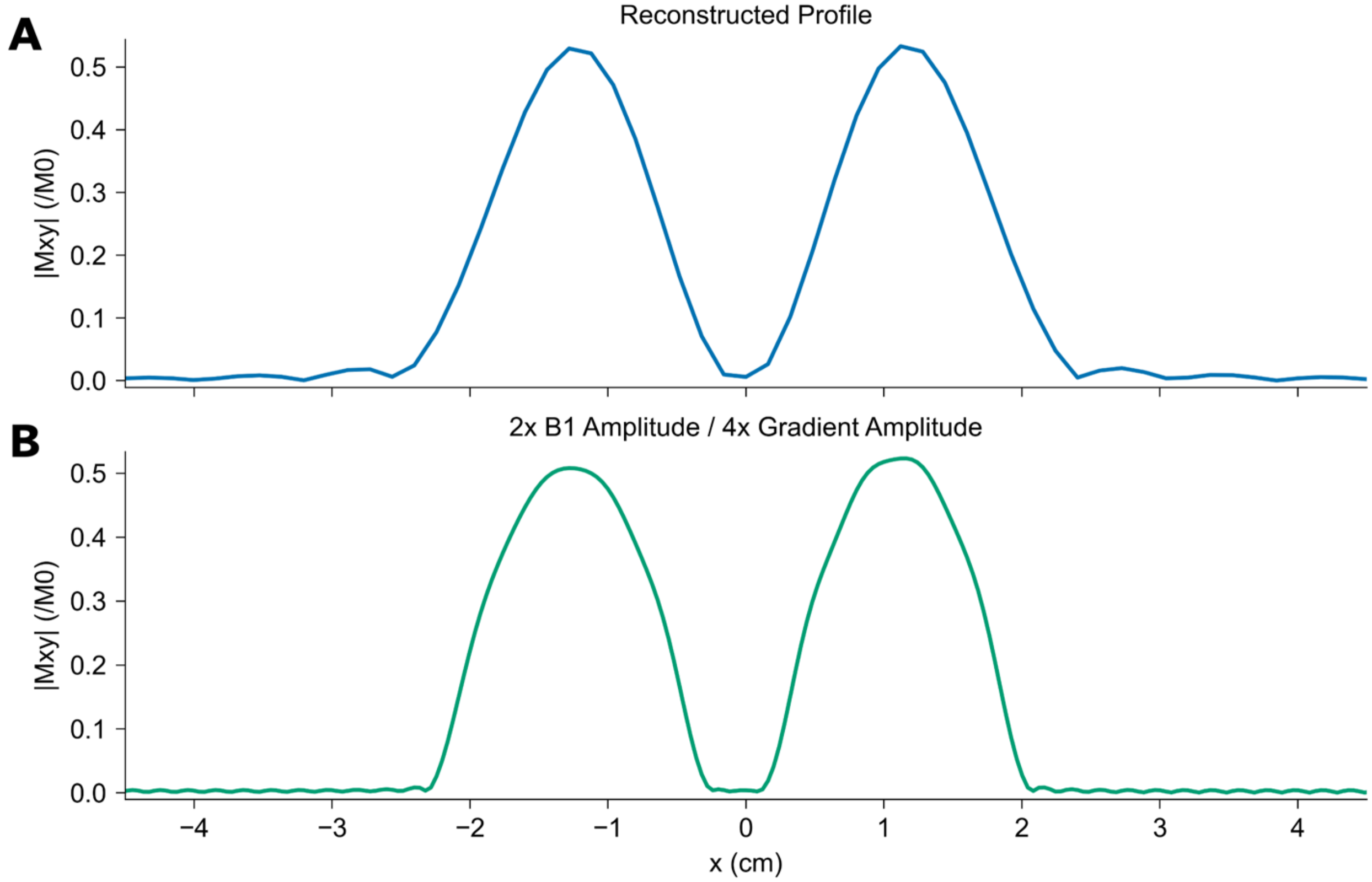

Supplementary Figure 12: *Two-ball phantom profiles reconstructed using (a) the experimental settings (B_1 amplitude and $\Delta\omega_{RF}$) and (b) using doubled B_1 amplitude corresponding to 4x effective gradient strength. The spatial resolution becomes finer, as expected with the stronger effective gradient strength.*

<u>Dependence of z-Signal Component on Excitation Flip Angle</u>

The size of the $M_z(x)\sin(\theta(x))\,e^{i\Delta\omega_{RF}t}$ term in the signal naturally depends on the excitation flip angle of the pulse sequence. The results shown so far used a 30° flip angle to illustrate the behavior and contribution of M_z to the received signal, but if a 90° flip angle is used, then $M_z(x) = 0$ and its highly oscillatory term should vanish. Supplementary Figure 13a confirms this, showing that when a 90° flip angle is used, the signal's offset and rapid oscillations

vanish, the peak at $\Delta\omega_{RF}$ in its spectrum vanishes (Supplementary Figure 13b), and the reconstructed signal increases as expected (Supplementary Figure 13c).

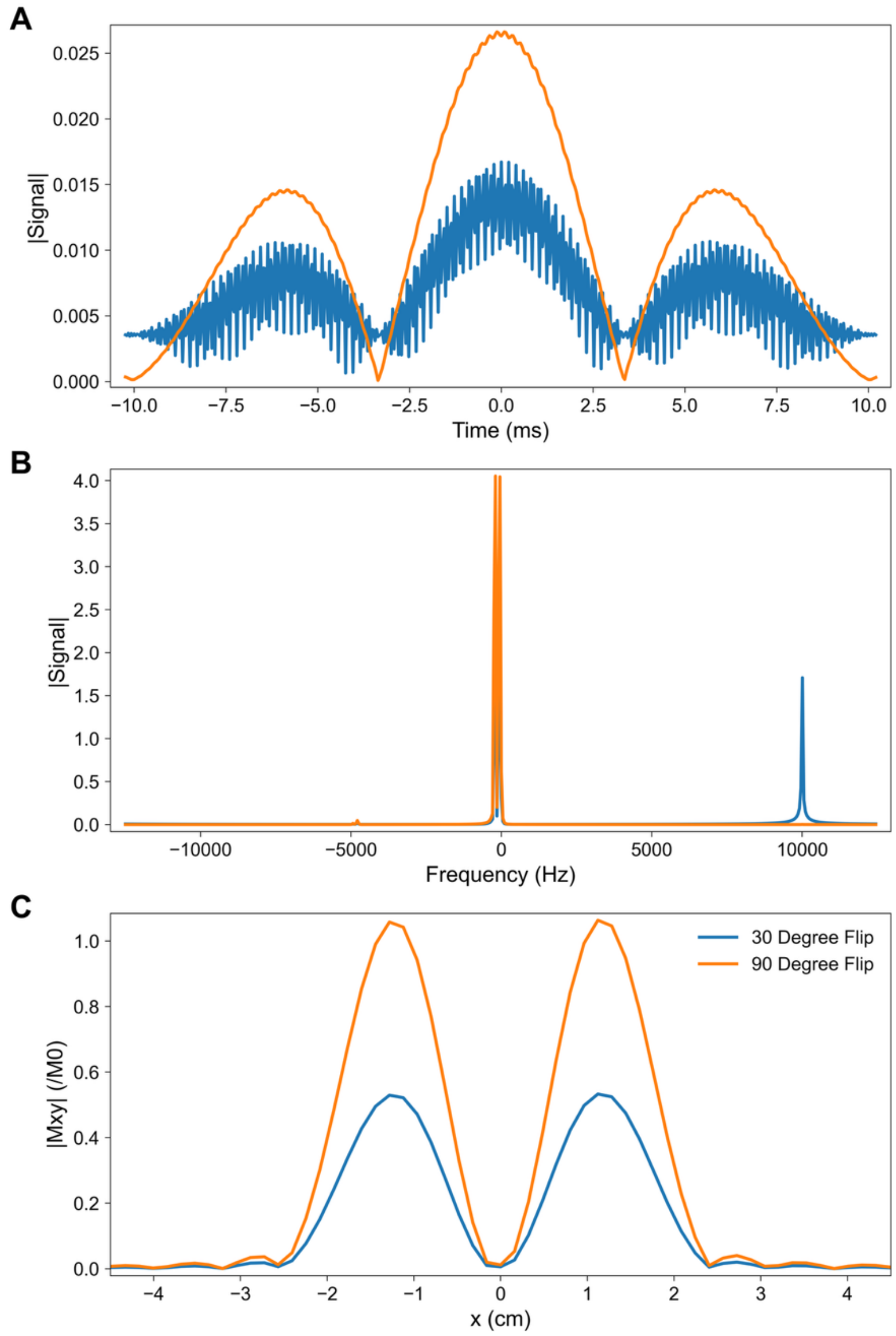

Supplementary Figure 13: *a) For a 90-degree excitation flip angle, the oscillatory M_z -dependent signal component vanishes, yielding a signal that is centered at zero. b) The signal component at* $\frac{\Delta\omega_{RF}}{2\pi}$ *= 10 kHz also vanishes at 90 degrees, and c) the reconstructed signal amplitude is higher, as expected.*

Chemical Shift

An important consideration in the design of frequency-encoded readouts is how much a pixel's signal will shift in the reconstructed image for a given resonance frequency offset (off-resonance) or chemical shift of the magnetization within the pixel. If we adopt the approximations that led to Equation 21, then Equation 24 relates a pixel's frequency offset of Δf Hz to its spatial shift of Δx meters in the reconstructed image as:

$$\Delta x = \frac{\Delta f}{\frac{\gamma}{2\pi} G} \tag{25}$$

$$= \frac{4\pi\Delta\omega_{RF}}{\gamma^2 c}\Delta f \tag{26}$$

Supplementary Figure 14 shows inverse FFT-reconstructed profiles from the simulated two-ball phantom with uniform off-resonance of Δf = 0 and 100 Hz, where the positions of the balls shifted by 1.6 cm in the 100 Hz-off-resonance case, as expected from Equation 26 with a $\frac{\Delta\omega_{RF}}{2\pi}$ = 10 kHz encoding pulse offset and c = 6.72 Gauss^2/meter, matching the experimental settings.

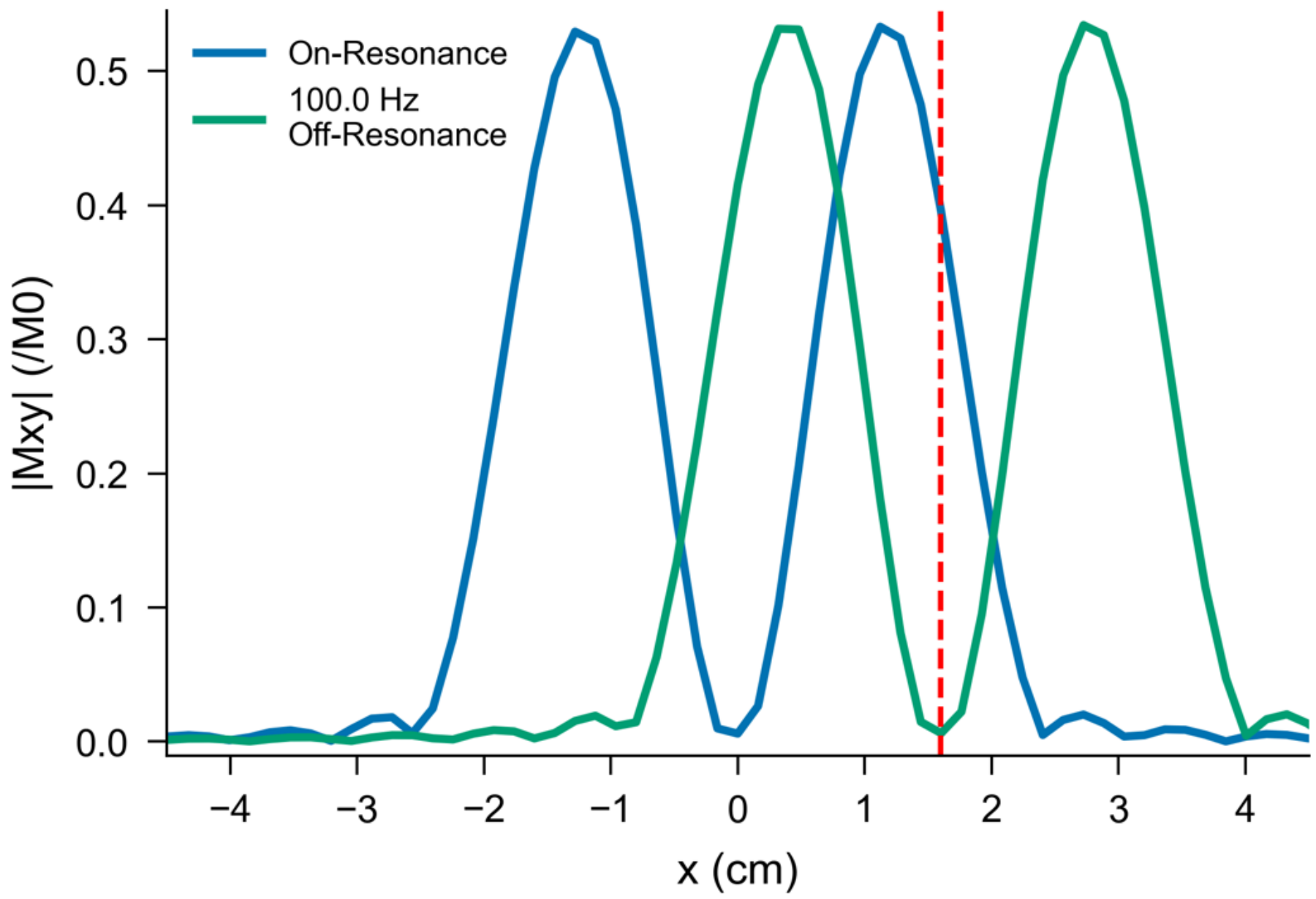

Supplementary Figure 14: *On-resonance reconstructed profile, versus a profile reconstructed from magnetization that was uniformly 100 Hz off-resonant. The expected shift was 1.6 cm, indicated by the red vertical line.*

<u>Supplementary References</u>

1. Srinivas, S. A., Martin, J. B., Vaughn, C. E. & Grissom, W. A. Linear Bloch–Siegert phase-encoded low-field MRI: RF coils, pulse sequence, and image reconstruction. *NMR Biomed.* e5245–e5245 (2024) doi:10.1002/NBM.5245.
