# Supplementary material for: Frequency-Encoded Magnetic Resonance Imaging with Dynamic Radio Frequency Field Gradients
Source: Commun Phys. Author manuscript; Available in PMC 2026 Aug 20. (PMC13488602; doi:10.1038/s42005-026-02686-5)
Supplement: Supplementary file 3 [file NIHMS2200620-supplement-Reviews.pdf]

nature portfolio

Peer Review File

Frequency-Encoded MRI with Dynamic Radio Frequency Field Gradients

Corresponding Author: Dr William Grissom

This file contains all reviewer reports in order by version, followed by all author rebuttals in order by version.

Version 0:

Reviewer comments:

Reviewer #1

(Remarks to the Author)
The authors should be commended on this very interesting development. The manuscript presents frequency encoding using RF gradients for the first time and this method could have important implications for portable MRI systems. The approach is nicely validated. I suggest changes to the manuscript structure and the inclusion of some additional information.

Major comments
1. The abstract and introduction state that there are two fundamental challenges that this manuscript addresses - 1) the mechanistic problem of needed to tip magnetization out of the transverse plane and 2) simultaneous transmit and receive. As a reader, I wanted the manuscript to then very explicitly tell me the solution to each problem, and it is not structured that way. The hardware implantation (#2 above) is well described in a dedicated section titled "Simultaneous Transmit and Receive for RF Frequency Encoding". However, the mechanistic solution (#1 above) is mostly addressed in the Supporting Information (as far as I could tell). A single paragraph (Page 8, Lines 192-200) glosses over one of the fundamental innovation. I suggest a separate heading on the mechanism of encoding without tipping magnetization, and bringing some of the key equations from the Supporting Information into the main text to explain (probably Equations 4-9).
2. Please add an animated video (not just static figures) of the magnetization to show how the frequency encoding mechanism works. I think this will improve the readability of the manuscript and clearly demonstrate the concept. Additionally, the difference between the standard magnetization equations in the rotating frame and the ones derived here should be made more explicit (this comparison is appropriate to keep in the supporting information).
3. The experiments performed are compelling to show that this approach works. Please include the phase information for Fig 6 in addition to the magnitude images.

Minor comments
4. Page 5, Line 125 – "without significantly tipping it out…" – the word "significantly" is misleading because it implies that some amount of intentional tipping is done via an RF pulse, and my understanding is that is not the case.
5. The text description of Figure 2A is long. It is important to include this information in order to highlight the parallels with Figure 2B, but it is standard to MRI readers and could be shortened with a reference.
6. Page 8, Line 198 – the phrasing "maximum tilt" is hard to interpret without reading the supporting information and should be elaborated or removed.
7. Figure 6 – "IR" (inversion recovery) is not defined anywhere
8. Extended data Figure 3 – the purpose of the negative lobe of the prephasor that is floating below the RF Gx Amplitude axis is unclear to me.

Reviewer #2

(Remarks to the Author)
The authors present a very interesting and creative approach to RF frequency encoding using Bloch-Siegert gradients enabled by a trnasformer-based simultaneous transmit and receive (STAR) system. I found the manuscript and the supporting information particularly engaging and well written. Below I list several questions and suggestions that I believe could further improve clarity and strengthen the work.

Transformer-based STAR system.

1. Ficure 3c demonstrates effective cancellation of the leaked transmit signal at the operating frequencies used in this study. It would be interesting to know whether the cancellation performance depends on frequency. For example, could the authors comment on or provide measurements of cancellation performance from on-resonance up to +- 100 kHz offest in regular frequency steps? This would help clarify the operational bandwidth of the proposed STAR system.
2. It would be helpful to clarify how often the cancellation calibration needs to be performed(especiaaly amplitude and phase adjustment for cancellation signal). For example, once the cancellation amplitude and phase are calibrated, can the same values be reused as long as the coil configuration remains unchanged, or is recalibration required when changing phantoms or for each scan? In addition, a more detailed description of how the optimal cancellation amplitude and phase are determined would improve reproducibility.
3. The manuscript states that the injuction transformer exhibits and insertion loss of 1.42 dB (line 254). However, this value is not clearly identifiable from Figure 3c. It may be useful to clarify how this value was measured. In addition, since 1.42 dB is not negligible, could the authors comment on whether this insertion loss alto applies when conventional B0 gradient encoding is used, or only in the STAR configuration?

RF coil design

4. The receive coil has a diameter of 3.5 cm, while two shperical phantoms have diameters of 2 cm. The RF gradient coil, however, is considerably larger. Could the authors clarify the rationale behind choosing this coil size?
5. For figure 4, could the authors specify the electromagnetic simulation method used to generate the simulated B1 map (e.g., Biot-Savart calculation, FDTD, FEM, or another approach)?
6. In extended data Figure 2, the receive coil appears to have a broader bandwidth than the RF gradient coil. However, quantitative information about the receive coil's Q-factor or bandwidth is not provided. Reporting these values would be helpful. Given that the RF gradient coil bandwidth appears approxiamtely 40 kHz based on its Q, and that the readout bandwidth is 25 kHz, it may be worth discussing whether a multi-turn receive coil could further improve SNR. If this consideration is not applicable, a brief explanation would be appreciated.
7. The manuscript mentions that the RF gradient coil is used to transmit both the excitation and the encoding pulses. A more detailed explanation of how this dual-frequency operation is achieved would be helpful. For example, is the coil bandwidth sufficiently wide to cover both 2.065 MHz and 2.075 MHz? Was a frequency-swapped pulse strategy used due to B1 inhomogeneity? Additionally, while the current experiments use relatively small phantoms compared to the coil size, it would be helpful to discuss whether this approach sould remain feasible for larger FOVs or whether a separate transmit coil would be preferable.

Scan parameters and encoding strategy.

8. Compared with the number of phase-encoding steps, substantially more samples are acquired along the frequency-encoding direction. Could the authors comment on the rationale for ths asymmetry?
9. Since Supporting Information indicates that increasing RF amplitude can effectively improve resolution, could the authors comment on whether increasing RF amplitude while reducing readout duration (and thus using fewer sample points) is feasible in this framework?
10. The peak RF power used for the encoding pulses was 12 W. could the authors clarify whether this value is limited by any consideration? Additionally, does the limit depend on operationg (encoding) frequency?

Supporting information.

11. It would be valuable if the Supporting Information (and/or main text) also more explicitly discussed the relationship between RF gradient strength, field-of-view (FOV), and spatial resolution, and how these parameters trade off with one another in RF frequency encoding.
12. In the Supporting Information simulations, the phantom geometry appears larger than the phantoms used in the experiments. It would be helpful either to include simulations using the same phantom size and placement as in the experiments, or to explicitly note that the simulated phantom differs in size, to avoid potential confusion.
13. Supporting Information suggests that increasing RF amplitude is more efficient for improving resolution than increasing B0 gradient amplitude. Given this, some discussion of why RF power was not increased beyond 12 W would be useful.

Minor/Editorila comments

14. line 408 appears to contain an empty set of parentheses.
15. In supporting information Figure 1, the coordinate axes are labeled x, y, and z. Based on the text, it may replace to x', y', and z' (however, z can remain unchanged). Additionally, including θ in the figure would aid interpretation.
16. In line 761, the vector notation on Mxy appears unnecessary and could be removed.

Version 1:

Reviewer comments:

Reviewer #1

(Remarks to the Author)
Thank you for your thorough response to my previous comments. I have no additional comments at this time.

Reviewer #2

(Remarks to the Author)
I appreciate the authors' careful revisions and responses to the reviewers' comments. The manuscript has been substantially improved, and the authors have addressed the previously raised concerns in a clear and satisfactory manner. The revisions have helped clarify the methodology and strengthen the overall presentation of the work.

The manuscript is well organized and the results are clearly presented and supported. I have no further major comments. In my opinion, the paper is now suitable for publication in its current form.

Open Access This Peer Review File is licensed under a Creative Commons Attribution 4.0 International License, which permits use, sharing, adaptation, distribution and reproduction in any medium or format, as long as you give appropriate credit to the original author(s) and the source, provide a link to the Creative Commons license, and indicate if changes were made.
In cases where reviewers are anonymous, credit should be given to 'Anonymous Referee' and the source.
The images or other third party material in this Peer Review File are included in the article's Creative Commons license, unless indicated otherwise in a credit line to the material. If material is not included in the article's Creative Commons license and your intended use is not permitted by statutory regulation or exceeds the permitted use, you will need to obtain permission directly from the copyright holder.
To view a copy of this license, visit https://creativecommons.org/licenses/by/4.0/

Response to Reviewers

We thank the reviewers for their careful evaluation of our paper and have incorporated their suggested changes in the revised manuscript. We feel that it is now much stronger and clearer thanks to their input.

Below, we respond to each point and outline the changes we have made. Where the points were not numbered, we numbered them to more easily refer to them. We use the notation "R1.3" to refer to "Referee #1, point #3" etc. We use black font for the referee comments, blue font for our responses. In the attached "marked" manuscript all the changes are shown in red font.

Referee 1

R1.1: The authors should be commended on this very interesting development. The manuscript presents frequency encoding using RF gradients for the first time and this method could have important implications for portable MRI systems. The approach is nicely validated. I suggest changes to the manuscript structure and the inclusion of some additional information.

The abstract and introduction state that there are two fundamental challenges that this manuscript addresses - 1) the mechanistic problem of needed to tip magnetization out of the transverse plane and 2) simultaneous transmit and receive. As a reader, I wanted the manuscript to then very explicitly tell me the solution to each problem, and it is not structured that way. The hardware implantation (#2 above) is well described in a dedicated section titled "Simultaneous Transmit and Receive for RF Frequency Encoding". However, the mechanistic solution (#1 above) is mostly addressed in the Supporting Information (as far as I could tell). A single paragraph (Page 8, Lines 192-200) glosses over one of the fundamental innovations. I suggest a separate heading on the mechanism of encoding without tipping magnetization and bringing some of the key equations from the Supporting Information into the main text to explain (probably Equations 4-9).

We thank the reviewer for the encouraging words and for pointing out this discrepancy. We agree that the mechanistic solution deserves equal prominence to the hardware solution in the main text. In response to this feedback and to R2.11, we have restructured the manuscript to address this concern by moving most of the Supporting Information signal equation material to the main body in two new sections titled "Signal Equation for RF Frequency Encoding with the Bloch-Siegert Shift" and "Relationship Between RF and B_0 Frequency Encoding" immediately following the "Frequency Encoding with RF Gradients" section and before the hardware section. We also moved Supporting Information Figure 1 to the main text (now Figure 3).

R1.2: Please add an animated video (not just static figures) of the magnetization to show how the frequency encoding mechanism works. I think this will improve the readability of the manuscript and clearly demonstrate the concept. Additionally, the difference between the standard magnetization equations in the rotating frame and the ones derived here should be made more explicit (this comparison is appropriate to keep in the supporting information).

Thank you for this suggestion. We have added Extended Data Video 1 which illustrates the motion of the magnetization in the off-resonant RF frame, as well as the signal in that frame and the Larmor frame, for two B_1 amplitudes. We have also added an explicit summary of the differences between the signal equations for Bloch-Siegert RF versus B_0 gradient encoding,

after showing the steps to connect them in the newly added "Relationship Between RF and B_0 Frequency Encoding" section.

R1.3: The experiments performed are compelling to show that this approach works. Please include the phase information for Fig 6 in addition to the magnitude images.

We thank the reviewer for this suggestion. We have added phase images as Extended Data Figure 7. This figure shows both magnitude and phase for all experimental conditions presented in Figure 7 (formerly Figure 6). The phase images confirm that RF frequency encoding generates phase information, with similar ranges of phase variation across the phantoms.

R1.4: Page 5, Line 125 – "without significantly tipping it out…" – the word "significantly" is misleading because it implies that some amount of intentional tipping is done via an RF pulse, and my understanding is that is not the case.

We appreciate the reviewer's careful attention to the language. We believe the phrasing accurately describes the physics: the off-resonant RF pulse does unavoidably tip the magnetization out of the transverse plane by a small angle θ during the readout. However, larger tilt angles reduce part of the transverse magnetization component (scaling as cos (θ)), directly reducing SNR. By operating in a regime where $\Delta\omega_{RF} \gg \gamma B_1$, we keep the tilt angle small and do not significantly tip it out of the transverse plane. We have read through the manuscript and adjusted wording where appropriate to ensure that we do not give the reader the false impression that no tipping at all occurs during Bloch-Siegert frequency encoding.

R1.5: The text description of Figure 2A is long. It is important to include this information in order to highlight the parallels with Figure 2B, but it is standard to MRI readers and could be shortened with a reference.

We agree and have condensed the description of the conventional GRE sequence while maintaining the essential comparison to RF frequency encoding.

R1.6: Page 8, Line 198 – the phrasing "maximum tilt" is hard to interpret without reading the supporting information and should be elaborated or removed.

We have removed this text. With the addition of the new section "Signal Equation for RF Frequency Encoding with the Bloch-Siegert Shift" (R1.1), the tilt angle is now properly defined and explained in context.

R1.7: Figure 6 – "IR" (inversion recovery) is not defined anywhere

Thank you for catching that. We have now defined "IR" at its first use in the main text.

R1.8: Extended data Figure 3 – the purpose of the negative lobe of the prephasor that is floating below the RF Gx Amplitude axis is unclear to me.

We apologize for the lack of clarity in Extended Data Figure 4 (formerly Figure 3). The visual appearance of the prephasor pulse in the RF G_x traces is better understood by viewing the detailed waveforms in Extended Data Figure 5b. The prephasor uses a frequency-swept pulse design where the frequency modulation peaks at the beginning and end cause the B_{eff} vector to start along the z-axis. This adiabatically sweeps the transverse magnetization before and

after the bulk of the phase encoding, which occurs during the flat central portion of the FM waveform[29]. This swept pulse design minimizes unwanted excitation compared to a constant frequency offset pulse. We have amended the figure captions to clarify this.

Referee 2

R2.1: The authors present a very interesting and creative approach to RF frequency encoding using Bloch-Siegert gradients enabled by a transformer-based simultaneous transmit and receive (STAR) system. I found the manuscript and the supporting information particularly engaging and well written. Below I list several questions and suggestions that I believe could further improve clarity and strengthen the work.
Transformer-based STAR system.
Figure 3c demonstrates effective cancellation of the leaked transmit signal at the operating frequencies used in this study. It would be interesting to know whether the cancellation performance depends on frequency. For example, could the authors comment on or provide measurements of cancellation performance from on-resonance up to +- 100 kHz offest in regular frequency steps? This would help clarify the operational bandwidth of the proposed STAR system.

We thank the reviewer for the kind words and for this excellent suggestion. We have added vector network analyzer (VNA) measurements characterizing the injection transformer's frequency response as Extended Data Figure 2. The measurement shows S_{21} between primary winding 1 and the secondary winding across a ±100 kHz bandwidth centered at the Larmor frequency (2.075 MHz).

The VNA measurement demonstrates that S_{21} remains flat across the 200 kHz span, confirming that the transformer's insertion loss is frequency-independent over this bandwidth. This validates that the performance shown in Figure 4c (formerly Figure 3c) at our 10 kHz encoding offset applies equally well across the full range of potential encoding frequencies. This clarification has been added in the Simultaneous Transmit and Receive for RF Frequency Encoding section.

R2.2: It would be helpful to clarify how often the cancellation calibration needs to be performed (especially amplitude and phase adjustment for cancellation signal). For example, once the cancellation amplitude and phase are calibrated, can the same values be reused as long as the coil configuration remains unchanged, or is recalibration required when changing phantoms or for each scan? In addition, a more detailed description of how the optimal cancellation amplitude and phase are determined would improve reproducibility.

We thank the reviewer for this important practical question. Once the cancellation amplitude and phase are calibrated for a particular experimental setup, the values can be reused across multiple scans without recalibration, provided the coil configuration remains unchanged. In our experiments with the current coil design, we found that loading changes (different phantoms) did not require recalibration due to the high geometric isolation between coils (>60 dB). However, recalibration was required when coil positions or cable routing changed. The high geometric decoupling minimizes load-dependent coupling variations, though this may vary with different coil designs where geometric isolation is lower.

Currently, we perform calibration manually by iteratively adjusting the amplitude and phase of the cancellation signal while monitoring the residual leaked signal and making sure it is lower than the dynamic range of the receiver, which takes approximately 5 minutes. For a given coil setup, this calibration needs to be performed only once. Future implementations

could employ automated calibration algorithms and more robust transformer designs to further improve stability and ease of use. To address these points, we added text to the Methods and Discussion sections.

R2.3: The manuscript states that the injection transformer exhibits an insertion loss of 1.42 dB (line 254). However, this value is not clearly identifiable from Figure 3c. It may be useful to clarify how this value was measured. In addition, since 1.42 dB is not negligible, could the authors comment on whether this insertion loss also applies when conventional B0 gradient encoding is used, or only in the STAR configuration?

We thank the reviewer for pointing out this lack of clarity. The insertion loss was measured separately using a vector network analyzer (VNA) rather than being derived from Figure 4c (formerly Figure 3c), which shows the cancellation performance during active signal acquisition. As described for R2.1, We have clarified the measurement method in the manuscript and added the VNA measurement as Extended Data Figure 2.

It is worth noting that insertion loss is a common feature of most simultaneous transmit and receive (STAR) and self-interference cancellation (SIC) techniques[39], which typically incorporate filtering, circulators, or other passive components that introduce some degree of signal attenuation. Future implementations could further reduce insertion loss through optimized receive coil design (increased number of windings), improved transformer core materials and winding configurations, or active coil decoupling strategies to reduce the leaked signal requiring cancellation. We have added this clarification in the Discussion section.

R2.4: RF coil design

The receive coil has a diameter of 3.5 cm, while two spherical phantoms have diameters of 2 cm. The RF gradient coil, however, is considerably larger. Could the authors clarify the rationale behind choosing this coil size?

We thank the reviewer for this question. The RF gradient transmit coil was originally designed for wrist or hand imaging[29], with dimensions (16 cm diameter, 34 cm length) suitable for that anatomy. The coil was optimized to produce a square-root B_1 field profile for Bloch-Siegert encoding. For this proof-of-concept study using small phantoms (2 cm diameter spheres), the coil's large size ensured highly uniform encoding gradients over the imaging volume. A smaller receive coil was used to maximize receive sensitivity for the small phantoms. The large size difference between transmit and receive coils also provided excellent geometric decoupling (>60 dB isolation), which was critical for minimizing the leaked signal requiring cancellation by the injection transformer.

For clinical translation, coil sizing would be optimized for the specific anatomy and application, with separate uniform-field excitation coils if needed for larger fields-of-view where B_1 inhomogeneity becomes significant. This clarifying text has been added to the Methods and Discussion sections.

R2.5: For figure 4, could the authors specify the electromagnetic simulation method used to generate the simulated B1 map (e.g., Biot-Savart calculation, FDTD, FEM, or another approach)?

We thank the reviewer for this question. The simulated B_1 maps in Figure 5a (formerly Figure 4a) were calculated using the Biot-Savart law. This quasi-static approximation is valid at our

operating frequency (2.075 MHz, wavelength λ ≈ 145 m) where the coil dimensions (34 cm length, 16 cm diameter) are much smaller than the wavelength (<<λ). We have added details on this to the Methods.

R2.6: In extended data Figure 2, the receive coil appears to have a broader bandwidth than the RF gradient coil. However, quantitative information about the receive coil's Q-factor or bandwidth is not provided. Reporting these values would be helpful. Given that the RF gradient coil bandwidth appears approximately 40 kHz based on its Q, and that the readout bandwidth is 25 kHz, it may be worth discussing whether a multi-turn receive coil could further improve SNR. If this consideration is not applicable, a brief explanation would be appreciated.

We apologize for the oversight. The receive coil has Q = 15.7 and -3dB bandwidth = 132 kHz, while the RF gradient transmit coil has Q = 51.8 and -3dB bandwidth = 40 kHz, confirming the reviewer's observation that the receive coil has substantially broader bandwidth.

The reviewer raises an excellent point about multi-turn receive coils. For this proof-of-concept demonstration, we chose a single-turn configuration for two main reasons. First, the single-turn design maximizes geometric decoupling from the transmit coil, achieving >60 dB isolation and minimizing the leaked signal requiring cancellation by the injection transformer as described in response R2.4. Second, the lower Q provides broader -3 dB bandwidth (132 kHz), which comfortably covers the readout bandwidth (25 kHz) and ensures uniform sensitivity across the encoded frequency range. Future implementations will employ multi-turn receive coils combined with optimized injection transformer designs to achieve the higher SNR necessary for clinical applications. This clarification has been added to the Methods and discussion sections.

R2.7: The manuscript mentions that the RF gradient coil is used to transmit both the excitation and the encoding pulses. A more detailed explanation of how this dual-frequency operation is achieved would be helpful. For example, is the coil bandwidth sufficiently wide to cover both 2.065 MHz and 2.075 MHz? Was a frequency-swapped pulse strategy used due to B1 inhomogeneity? Additionally, while the current experiments use relatively small phantoms compared to the coil size, it would be helpful to discuss whether this approach should remain feasible for larger FOVs or whether a separate transmit coil would be preferable.

We thank the reviewer for this question. The RF gradient coil bandwidth (40 kHz, ±20 kHz from center) was sufficiently wide to cover all required frequencies. The coil was tuned and matched to the Larmor frequency (2.075 MHz) with S_{11} = -21.5 dB. At the encoding frequency offsets of ±10 kHz (2.085 MHz for the prephasor pulse and 2.075 MHz for the readout pulse), the coil maintained adequate matching with S_{11} = -6.6 dB and -5.9 dB, respectively. No frequency-swapped pulse strategy was used.

Regarding B_1 inhomogeneity: for this proof-of-concept study with gradient-echo pulse sequences, the RF gradient coil's B_1 variation across the imaged ball phantoms was not so great as to prevent clear visualization of the phantoms. As discussed in R2.4, for larger fields-of-view approaching the coil dimensions, a separate uniform-field transmit coil for excitation may be preferable to avoid excitation flip angle inhomogeneity, and the gradient coil would be dedicated to encoding only. These clarifications have been added to the Validation and Discussion sections.

R2.8: Scan parameters and encoding strategy.
Compared with the number of phase-encoding steps, substantially more samples are

acquired along the frequency-encoding direction. Could the authors comment on the rationale for this asymmetry?

We thank the reviewer for this question. The asymmetric sampling matrix (512 frequency-encode points × 34 phase-encode steps) was chosen for two reasons. First, acquiring many points along the frequency-encoding dimension is inexpensive in terms of scan time, and the correspondingly large FOV along that dimension (proportional to the readout bandwidth) ensures that the off-resonant M_z signal component, which is displaced by $\Delta\omega_{RF}$ from the image center, does not alias into the reconstructed image. Second, using only 34 phase-encode steps reduced the total acquisition time per average to 15.19 seconds while providing adequate resolution to separate the two phantoms along the phase-encode dimension. This rationale has been added to the Validation section.

R2.9: Since Supporting Information indicates that increasing RF amplitude can effectively improve resolution, could the authors comment on whether increasing RF amplitude while reducing readout duration (and thus using fewer sample points) is feasible in this framework?

We thank the reviewer for this insightful question. Yes, increasing RF amplitude to strengthen the gradient and enable shorter readouts is feasible in principle.

For this proof-of-concept demonstration, we chose conservative parameters (12 W peak RF power, 20.48 ms readout) to balance multiple considerations. Higher B_1 increases the tilt angle θ, reducing SNR. Additionally, to simplify STAR cancellation, the constant-frequency readout pulse did not employ the adiabatic frequency sweeps used in the prephasor pulse, so higher B_1 values cause unwanted excitation during the readout, as illustrated in Extended Data Figure 9 (formerly Figure 7) for the prephasor. The pre-emphasis compensation for the leaked transmit signal (Extended Data Figure 7) also becomes more challenging with higher RF power. Our goal was to demonstrate the feasibility of RF frequency encoding rather than to optimize scan parameters.

Future optimized implementations could explore this trade-off space more fully, using swept-frequency readout pulses to minimize unwanted excitation, improved transformer isolation, and active decoupling to enable higher RF power and faster acquisitions while maintaining image quality. This clarification has been added to the Discussion section.

R2.10: The peak RF power used for the encoding pulses was 12 W. could the authors clarify whether this value is limited by any consideration? Additionally, does the limit depend on operating (encoding) frequency?

We thank the reviewer for this question. The 12 W peak RF power was not limited by fundamental constraints but was chosen for this proof-of-concept demonstration to balance encoding performance with practical considerations. The primary limitations at higher power would be: (1) increased tilt angle θ reducing SNR, (2) unwanted excitation from the constant-frequency readout pulse (as discussed in R2.9 and shown in Extended Data Figure 9), and (3) increased leaked signal amplitude requiring better cancellation performance from the injection transformer.

The RF power requirement does depend on the encoding frequency offset. For a given desired gradient strength, lower encoding offsets ($\Delta\omega_{RF}$) require less RF power since the Bloch-Siegert shift scales as $B_1^2/\Delta\omega_{RF}$. However, operating too close to resonance compromises the transformer's ability to isolate the encoding signal from the much weaker proton signal. Our

choice of 10 kHz offset balanced efficient encoding (requiring reasonable RF power) with practical isolation requirements. This has been added to the Discussion section.

R2.11: It would be valuable if the Supporting Information (and/or main text) also more explicitly discussed the relationship between RF gradient strength, field-of-view (FOV), and spatial resolution, and how these parameters trade off with one another in RF frequency encoding.

Thank you for this suggestion. In response to this suggestion and R1.1, we have added these relationships to the "Relationship Between RF and B_0 Frequency Encoding" section in the manuscript.

R2.12: In the Supporting Information simulations, the phantom geometry appears larger than the phantoms used in the experiments. It would be helpful either to include simulations using the same phantom size and placement as in the experiments, or to explicitly note that the simulated phantom differs in size, to avoid potential confusion.

Thank you for catching this error: the simulations used phantoms of the same size as the experiment, but in Figure (a) we mislabeled the x-axis, making them appear much larger. We have fixed this.

R2.13: Supporting Information suggests that increasing RF amplitude is more efficient for improving resolution than increasing B0 gradient amplitude. Given this, some discussion of why RF power was not increased beyond 12 W would be useful.

As discussed in responses R2.9 and R2.10, higher RF power was limited in this experiment by increased tilt angle (reducing SNR), unwanted excitation from the constant-frequency readout pulse, and increased leaked signal requiring better cancellation. Our priority for this proof-of-concept work was demonstrating first-ever RF frequency encoding rather than parameter optimization.

R2.14: line 408 appears to contain an empty set of parentheses.

Thank you for your careful reading. The notation intlinprog() follows the standard convention for referring to function names in MATLAB, where the empty parentheses indicate that it is a callable function. We have changed the font to Courier to make it clearer that this is code notation.

R2.15: In supporting information Figure 1, the coordinate axes are labeled x, y, and z. Based on the text, it may replace to x', y', and z' (however, z can remain unchanged). Additionally, including θ in the figure would aid interpretation.

Thank you for catching this discrepancy. Supporting Information Figure 1 has been moved to the main text as Figure 3 (R1.1). We have updated the axis labels to x', y', and z, and have added the tilt angle θ between the z-axis and the B_{eff} vector in the right panel (High B_1 case).

R2.16: In line 761, the vector notation on Mxy appears unnecessary and could be removed.

Thank you for noting this, we have removed it.
